# Bioethical implications of end-of-life decision-making in patients with dementia: a tale of two societies

**DOI:** 10.1007/s40592-020-00112-2

**Published:** 2020-04-25

**Authors:** Jaime D. Mondragón, Latife Salame-Khouri, Arnoldo S. Kraus-Weisman, Peter P. De Deyn

**Affiliations:** 1 Department of Neurology, University of Groningen, University Medical Center Groningen, PO Box 30001, 9700 RB Groningen, The Netherlands; 2 Alzheimer Center Groningen, University of Groningen, University Medical Center Groningen, Groningen, The Netherlands; 3 Facultad de Medicina, Unidad de Posgrado, Universidad Nacional Autónoma de México, Mexico City, Mexico; 4 Department of Internal Medicine, The American British Cowdray Medical Center, Mexico City, Mexico; 5 Laboratory of Neurochemistry and Behavior, Institute Born-Bunge, University of Antwerp, Antwerp, Belgium

**Keywords:** Anosognosia, Bioethical, Dementia, Decision-making, Euthanasia

## Abstract

End-of-life decision-making in patients with dementia is a complex topic. Belgium and the Netherlands have been at the forefront of legislative advancement and progressive societal changes concerning the perspectives toward physician-assisted death (PAD). Careful consideration of clinical and social aspects is essential during the end-of-life decision-making process in patients with dementia. Geriatric assent provides the physician, the patient and his family the opportunity to end life with dignity. Unbearable suffering, decisional competence, and awareness of memory deficits are among the clinical considerations that physicians should incorporate during the end-of-life decision-making process. However, as other societies introduce legislature granting the right of PAD, new social determinants should be considered; Mexico City is an example. Current perspectives regarding advance euthanasia directives (AED) and PAD in patients with dementia are evolving. A new perspective that hinges on the role of the family and geriatric assent should help culturally heterogeneous societies in the transition of their public health care policies regarding end-of-life choices.

**Electronic supplementary material:**

The online version of this article (10.1007/s40592-020-00112-2) contains supplementary material, which is available to authorized users.

## Introduction

It was the best of times, it was the worst of times, it was the age of wisdom, it was the age of foolishness, it was the epoch of belief, it was the epoch of incredulity, it was the season of Light, it was the season of Darkness, it was the spring of hope, it was the winter of despair, we had everything before us, we had nothing before us, we were all going direct to Heaven, we were all going direct the other way—in short, the period was so far like the present period, that some of its noisiest authorities insisted on its being received, for good or for evil, in the superlative degree of comparison only. Charles Dickens, A Tale of Two Cities.

### Definitions and legal due care criteria for PAD

Physician assistance in dying or physician-assisted death (PAD) refers to physician-assisted suicide (PAS) and euthanasia (Dresser 2017). PAS is when a physician, upon patient request, provides a self-administered lethal medication, while meeting the legal due care criteria for PAD (Haan 2002). In contrast, euthanasia alludes to the administration of a lethal medication by a physician at the request of a patient (Haan 2002). While both euthanasia and PAS are legal in the Netherlands, Luxemburg, and Canada, euthanasia is the only legal form of PAD in Belgium and Colombia (Emanuel et al. 2016), while Victoria, Australia, permits PAS and euthanasia is also allowed for people for whom PAS is not viable because they cannot take the medications themselves to end their life. Furthermore, PAS is the only legal form of PAD, which is referred to as ‘Aid in dying’, in Oregon, Washington, Montana, Vermont, California (Emanuel et al. 2016), Colorado, New Jersey, Maine, Hawaii and Washington D.C.; while in Switzerland assisted suicide in a non-medical context is allowed. Since most countries have adopted a version similar to the Dutch due care criteria, we have opted to discuss these. The due care criteria for PAD in the Dutch Euthanasia Act are: (1) the attending physician has come to the conviction that the request from the patient is voluntary and well-considered; (2) the attending physician has come to the conviction that the suffering of the patient is unbearable and without prospect of improvement; (3) the physician has informed the patient about his situation and prospects; (4) the patient and physician must concur that there are no more reasonable alternatives to relieve suffering; (5) the physician has consulted an independent physician; (6) the physician has terminated the patient’s life or assisted with suicide with due medical care and attention (Beaufort and Vathorst 2016; RTE Annual Reports 2004; Snijdewind et al. 2015). For an abridged legal framework of physician-assisted death, we refer the reader to the Supplementary Material.

In Mexico, PAD is illegal. Euthanasia is considered a homicide and carries a 10-year to a 14-year prison sentence, while PAS is classified as an assisted homicide and carries a 2-year to a 5-year prison sentence (Río et al. 2017). In 2008, Mexico City approved a law (i.e. “Ley de Voluntad Anticipada” or Advanced Directives Law) that legalizes advance health care directives (AHD), allowing citizens to establish a medical directive regarding the continuation or termination of treatments that extend life under conditions that the subject deems not worth living (Secretaría de Salud de la Ciudad de México 2008). This law absolves the physician who discontinues the treatments aimed at extending the life of any legal prosecution. Approximately half (i.e. 15 out of the 31) of the states in Mexico have an AHD law similar to that of the country’s capital (Río et al. 2017). Along with the AHD law, the General Health Law (i.e. “Ley General de Salud”) establishes that patients with terminal illnesses must be offered palliative care measures (Salud 2007). Among the palliative care options, the patient may receive palliative sedation. Even though PAD is illegal, the patient has the right to request irreversible deep sedation; meanwhile, the physician is protected by law (Río et al. 2017). For a thorough analysis of the cultural and political attitudes, as well as current and future perspectives toward PAD in Mexico we refer the reader to the text by del Río (Río et al. 2017) and the book by González-Huerta et al. (2017).^1^ Nevertheless, due to poverty, just a small portion of inhabitants can receive palliative options of good quality. As of January 30, 2017, the constitution of Mexico City under article 11 incorporates the right of personal auto-determination, and the right to live with dignity; which in its own right includes the right to pursue death with dignity (Asamblea Constituyente Ciudad de México 2017).

### Advance euthanasia directive and geriatric assent (vide infra)

Euthanasia and PAS are complex topics that promote debate in both public opinion and the medical profession around the world. In traditional-religious oriented cultures people consider religion, family and deference to authority as important values; conversely, they reject divorce, abortion, suicide, and euthanasia (Heide 2013). The strategy for the evaluation regarding decision-making capacity can be simplified into five steps according to Moberg and Rick (Moberg and Rick 2008), (1) clarification of the referral question and determination of the patient’s competence, (2) planning, cultural assessment and ethical considerations according to the topic, (3) assessment, (4) communication of the results, and (5) recommendation of any additional evaluations or interventions (Moberg and Rick 2008). Adding another level of complexity to this debate is the opinion and influence that religion plays in public policymaking in Latin America (Río et al. 2017). While public opinion polls in the United States suggest a decline in support regarding euthanasia from a peak of 75% in 2005 to 64% in 2012, in Central and Eastern Europe there has been no increase and even a decrease in acceptance, also due to religious movements; however, in Western Europe support for euthanasia increased (Emanuel et al. 2016). The perspectives of the general population, surveyed to date, in Mexico (i.e. adults 18–49 years of age, middle or high income and Catholics) regarding their acceptance of euthanasia oscillates between 60 and 65% (Río et al. 2017; Muñoz 2005). Acceptance of PAD from the general population and the medical profession has been on the rise (Río et al. 2017). Complete acceptance of PAD hinges on raising awareness and educating both the public as well as the medical profession; nonetheless, since more than fifty million people in Mexico live in poverty, topics such as euthanasia are not part of public discourse.

Until now, requests for advance euthanasia directives (AED) must describe the point at which death should occur (Río et al. 2017); however, this decision as difficult as it might be, must be discussed between the patient and the physician. The due care criteria also apply when the patient formulates an AED (RTE Annual Reports 2004). In the Netherlands although not a legal requirement, it is advised that the directive be updated on regular intervals and should define as clearly as possible the circumstances in which PAD is the preferred option (RTE Annual Reports 2004). Furthermore, the committees recommend the physician to record details about the general discussion regarding the patient’s end-of-life wishes to provide solid arguments regarding the joint PAD decision-making process (RTE Annual Reports 2004). The clinician must consider that by the time a patient meets his/her pre-established AED criteria they probably do not remember their earlier request and may show no signs of suffering (Dresser 2017). In this regard, Belgian law stipulates that the patients who requested an AED, in *compos mentis* and the presence of two witnesses, must be no longer conscious (Landry et al. 2015) and the AED is only applicable in cases of unconsciousness. In this context, unconsciousness is a complex clinical term and an even more intricate neurobiological phenomenon. Unconsciousness could refer to the clinical state of decreased wakefulness (i.e. decreased brain stem function), decreased awareness of the patient’s surroundings (i.e. decreased cortico-thalamic network activation) or even lack of awareness of memory deficits (i.e. anosognosia, decreased activation of default mode network and middle cortical structures).^2^ To avoid future problems the Dutch RTEs advise the physicians who are discussing euthanasia with their patients to draw up a written directive in good time; furthermore, the RTEs recommend that the physicians discuss the content and implications of such directive with the patient, advising that “the more specific the euthanasia directive, the firmer the basis for the attending physician’s decision and the committee’s assessment (RTE Annual Reports 2004). Furthermore, although not a legal requirement, the RTE suggests that the directive should be handwritten by the patient, for clarification purposes, thus describing of the circumstances in his own words.

Advance euthanasia directives and PAD requests hinge on an intimate patient-physician relationship, thus geriatric assent can provide an adequate basis for a shared decision process. Patients with dementia aren’t, usually, asked about their opinion in the informed consent process. Although, the patient might have limited decision-making capacity, nonetheless, they are still capable of expressing their preferences. Geriatric assent refers to the process of considering the preferences of an older and cognitively impaired patient in decision-making; furthermore, by knowing the patients’ preferences, physicians that practice geriatric assent, protect patients' autonomy and the decisions that will be made about their own life and health (Molinari et al. 2004). Geriatric assent is a process that balances the limited cognitive capacities of a cognitively impaired patient with the priorities of the family members to maintain involvement in health care decisions (Molinari et al. 2006). The four step-process for promoting geriatric assent include: (1) identifying the patient’s long-standing values and preferences; (2) assessing plans of care in terms of biopsychosocial safety and independence along with the patient's values and preferences; (3) protecting remaining autonomy; and (4) cultivating the professional virtues of steadiness, self-effacement, and self-sacrifice when making decisions that risk the patient's future health and safety (Coverdale et al. 2006). Geriatric assent is best accomplished with a multidisciplinary team that encourages family involvement and that understands the patient’s health-related cultural context (Molinari et al. 2006). Geriatric assent provides the physician, the patient, and his family the opportunity to end life with dignity. Death with dignity should always be the end result of PAD; taking into consideration the patient’s psychological needs and emotional state, the patient’s susceptibility for depression and demoralization, the patient’s competence and ability to process information, and the caregivers’ influence and psychological situation (Cohen-Almagor 2016).

### Objectives

The primary aim of this article is to build upon two bioethical perspectives (i.e. Flemish and Dutch versus Mexican perspective) for clinical decision-making regarding physician-assisted death for patients with dementia. To achieve this we provide contrasting, but supplementary bioethical perspectives regarding the decision-making process in PAD for patients with dementia from Belgium, the Netherlands, and Mexico. We will engage in a dialectic exercise, where the contrasting perspectives on PAD for patients with dementia will serve as the platform to discuss the bioethical attitudes and clinical approaches to assist the end-of-life decision-making process. A secondary aim of this work is to serve as an update of the recent changes regarding the outlook of end-of-life perspectives toward PAD and AED in patients with dementia, as well as to propose a new perspective that focuses on geriatric assent and the role of the family in societies that have a family-physician medical consensus approach to end-of-life decision making.

## Bioethical implications of end-of-life decision-making in dementia

End-of-life decision-making in patients with dementia is a complex topic. Careful consideration of clinical and social aspects is essential in the end-of-life decision-making process in patients with dementia. Unbearable suffering and decisional competence are two clinical topics that have emerged as part of the discussion when evaluating the due care process in cases reporting PAD in dementia in RTE annual reports during the last 5 years. Awareness of memory deficits has posed a serious challenge for clinicians attempting to assess the validity of an AED. As more societies are introducing laws legalizing PAD, more challenges arise. The challenges that Mexico City will face in the upcoming years will provide further insight into the complex relationship between medical issues that have ample social consequences. In the next section, the authors will present current perspectives and a new perspective that hinges on the role of the family and geriatric assent. For perspectives opposing PAD and AED in patients with dementia, current perspectives of PAD and palliative care for patients with dementia in Mexico, and the PAD cases involving patients suffering from dementia in the annual Dutch review committee reports, we refer the reader to the supplementary material.

### End-of-life outcomes in patients with dementia in Belgium and the Netherlands

Belgium and the Netherlands have been at the forefront of legislative advancement and progressive societal changes concerning the perspectives toward PAD. The first case of PAD for a patient with dementia reported by the Dutch RTE Annual Report was in 2004 and the first case with advanced dementia was notified in 2011. Data on PAD among patients with dementia in the Netherlands indicates an increase of 49 cases in 2009 to 97 cases in 2013 and 84 in 2014 (Beaufort and Vathorst 2016). In comparison, the proportion of PAD reported cases with dementia in Belgium has gradually increased accounting for 0.25% (5 cases) from 2002 to 2007 reaching 0.83% (57 cases) from 2008 to 2013 (Dierickx et al. 2017).

The Dutch regional committees began to specifically report PAD in patients with dementia from 2009; reporting only seven cases from 2002 to 2006, 86 from 2007 to 2011, 470 from 2012 to 2016, and 169 cases in 2017 (i.e. 166 early dementia and 3 advanced or very advanced stage of dementia). All the attending physicians, when notifying a PAD involving a patient with dementia, acted in accordance with the laws of due care from 2002 to 2011; however, from 2012 to 2016, three cases involving dementia patients did not comply with the due care criteria. The three cases involved patients with an advanced stage of Alzheimer’s disease (AD). Requests for PAD, for all diseases, are granted by physicians at a rate of 25–45% in the Netherlands (Snijdewind et al. 2015; Jansen-van der Weide et al. 2005; Onwuteaka-Philipsen et al. 2012; Maas et al. 1996). Among the five medical conditions (i.e. somatic, psychological, somatic and psychological, cognitive decline, and tired of living) reported where there was a request for PAD, cognitive decline had the highest rate of granted requests at 37.5% compared to an overall granted rate of 25.1% for the five medical conditions (Snijdewind et al. 2015). Additionally, requests for PAD of patients with cognitive decline were the second-lowest with 35.7%; meanwhile, somatic medical conditions had the lowest rejection rate, with 34% and an overall rejection rate of 46.5% (Snijdewind et al. 2015). Analogously, a Belgian study found that 48% of the request for euthanasia in 100 consecutive psychiatric patients requesting euthanasia was granted; although the study did not include dementia patients, 90% of the patients had more than one psychiatric disorder, with depression (i.e. 58%) and personality disorders (i.e. 50%) being the most frequent cause of unbearable suffering (Thienpont et al. 2015). The reader may consult the article by Emanuel and colleagues (2016) for a comprehensive review of the attitudes and end-of-life outcomes regarding PAD around the world.

The first Belgian case referred to the public prosecutor occurred in 2015 (i.e. physician Marc Van Hoey, involving a patient with untreatable depression); meanwhile, in the Netherlands, 75 cases have been sent to the public prosecutor between 2002 and 2015, yet neither the Dutch or Belgian physicians have been prosecuted (Emanuel et al. 2016). The great majority of cases involving PAD in patients with dementia from 2007 to 2011 have involved early-stage patients who still understood their illness and the symptoms of loss of orientation and personality (Legemaate and Bolt 2013). Interestingly, in cases involving an AED for patients with advanced dementia, the attending physicians tend to disregard the directive and base their decisions on the current situation (i.e. basing their decision on the moral concept of beneficence rather than the patient’s autonomy) (Hertogh et al. 2007).

### Clinical considerations regarding AEDs for patients with dementia

#### Unbearable suffering in patients with dementia

Unbearable suffering is a subjective attribute of primary importance when the decision to proceed with a PAD in a patient has to be made. Furthermore, the clinical determination of unbearable suffering in a patient with dementia who may no longer be able to communicate is a complex topic. A physician must be able to interpret the body language and verbal responses from the patient (Legemaate and Bolt 2013). In advanced stages of dementia, the physician must indirectly infer the patient’s suffering from behavioral cues related to loss of brain function such as loss of control, anxiety, mood swings, and helplessness; additionally, the patient may also suffer from behavioral and psychological symptoms of dementia (BPSD) and neuropsychiatric symptoms (NPS), such as self-harm, stereotypic behavior, hallucinations, among others. A physician must act with due diligence when the patient is in the early stages of AD and request a PAD. In such cases, the RTEs recommend consulting with at least one specialist, a neurologist, psychiatrist or geriatrician, in addition to the independent physician (RTE Annual Reports 2004).

The Royal Dutch Medical Association (RDMA) position paper regarding the role of the physician in PAD, considers that psychosocial or existential suffering is part of the medical domain; furthermore, the RDMA states that vulnerability (e.g. loss of function, loneliness, and loss of autonomy) should be factored into the PAD decision-making process (Legemaate and Bolt 2013). In 2006, the RTE Annual Report recommended that PAD for patients with dementia should be “reserved for people with the unusual and painful combination of incipient dementia and very clear awareness of their disease” (RTE Annual Reports 2004). The committees recommend the physicians take into account the stage of the disease and other specific circumstances of the case before reaching a decision; furthermore, during the early stages of dementia, the treating physician should consult one or more experts in addition to the independent physician (RTE Annual Reports 2004). For a patient with dementia, the idea of consciously experiencing the unavoidable physical and mental decline, alongside with the fear of increasing loss of control over their mental faculties and a perceived sense of dependence as humiliating, may be sufficient to classify the patient’s suffering as unbearable (RTE Annual Reports 2004). Some researchers argue that patients with dementia adjust actively to their disease through emotional and problem-oriented strategies (Marwijk et al. 2007). For a discussion about bioethical concepts and philosophical arguments regarding PAD in patients with dementia, the authors refer the reader to the work by de Beaufort and van de Vathorst (2016) for insight in the concepts of voluntariness and suffering (de Beaufort and van de Vathorst 2016). The RTEs recommend that the attending physician must be able to convey effectively in his report when notifying a PAD that the suffering by the patient was unbearable (i.e. the RTEs refer to “unbearable suffering”). If the unbearable suffering criterion is not met during the independent physician’s evaluation, the RTEs recommend the attending physician should request another evaluation based on the estimation for further deterioration provided by the independent physician or if the clinical picture suddenly worsens (RTE Annual Reports 2004). For the attending physician to determine effectively if the patient requesting a PAD is undergoing unbearable suffering, he must be able to not only empathize with the patient’s situation but also with his point of view (e.g. life-history and values). To this extent, RTEs emphasize that the attending physician must be able to justify that the suffering was unbearable to that specific individual.

#### Decisional competence

According to the current legal framework in Belgium and the Netherlands, a voluntary and well-considered AED request drawn up in *compos mentis* represents the patient’s wishes in the case where the patient is not able to give his consent for the PAD. However, the due care criterion stipulating unbearable suffering with no prospect of improvement remains fully applicable (RTE Annual Reports 2004). Two types of AED based on the future patient’s treatment wishes exist. The first, a negative directive, where treatments are withheld in specific circumstances; secondly, a positive directive, describing the treatments or actions that the patient would like to be carried out in certain conditions (Hertogh et al. 2007). While negative advance directives are binding according to the Dutch Medical Treatment Act, positive advance directives cannot be imposed on the attending physician (Hertogh et al. 2007). Clinical assessment of competency can be comprised of a detailed and comprehensive interview, a targeted neuropsychological evaluation, functional ability assessment, and a review of the legal standards (Moberg and Rick 2008). Usually, this assessment of the patient’s decision-making capacity is an implicit part of the physician–patient interaction and made without the patient’s awareness (Appelbaum and Grisso 1988). Optimally, the patient and physician should have a professional but close relationship, one that can only be attained throughout the years of personal interaction. Capacity may be defined as a threshold requirement for a person to make an autonomous decision (Moye et al. 2013). While “capacity” refers to a clinical concept determined by a physician or health professional (Moye et al. 2013; Jacoby and Steer 2007), “competency” alludes to the ability of an individual to make decisions and is assessed by a legal professional (Moberg and Kniele 2006). The concept of competence alludes to four abilities: (1) ability to communicate choices; (2) understanding relevant information; (3) appreciating the situation and its consequences; (4) manipulating information rationally (Appelbaum and Grisso 1988). Evaluation of treatment consent capacity is relevant to the decision-making process regarding a PAD request, while evaluation of testamentary capacity is appropriate if the patient desires to retract an AED. The ideal assessment of testamentary capacity should include “a general psychiatric evaluation, a clinical interview with [the] observation of [the] patient’s functional abilities, a set of neuropsychological tests, an evaluation of the patient’s functional abilities, and the consideration of the current legal framework” (Voskou et al. 2018). Patients with dementia may be impaired in their capacity to consent to medical treatment abilities; furthermore, as dementia progresses, so does the consent impairment (Moye and Marson 2007). While decision-making abilities vary across individuals, competent individuals must possess the ability to choose independently, understanding the important information relevant to their choice (Dresser 2017). The nature of the request for PAD or AED is voluntary, and be carefully considered by the patient (RTE Annual Reports 2004). Patients seeking PAD must understand the range of patients’ experiences with dementia (Dresser 2017).

#### Awareness of memory deficits

Anosognosia is the lack of awareness of motor, visual or cognitive deficits in patients with neurological diseases (Langer and Levine 2014) and involves complete loss of knowledge of one’s impaired neurological or neuropsychological functions (Prigatano 2014). Many models of awareness have been developed to explain anosognosia, Cognitive Awareness Model (CAM) (Morris and Mograbi 2013), Autobiographical Memory in Alzheimer’s disease (AMAD) (Haj et al. 2015), and Feeling of Knowing (FOK) (Cosentino and Stern 2005; Schnyer et al. 2004) among others, yielding many approaches and perspectives regarding the unawareness of memory deficit phenomenon. Interestingly, clinical data associate anosognosia to different dementias, eventually exhibited by nearly all persons with dementia (Wilson et al. 2016).

The validity of AED in patients with advanced dementia has been questioned since many patients at this stage present anosognosia and may not meet the unbearable suffering requirement of the due care criteria (Hertogh et al. 2007; Hertogh 2009); nonetheless, suffering in AD is a complex physiological and psychological phenomenon. The degree of awareness of memory deficits in AD is associated with depressed mood, as increased awareness is correlated to increased depressive symptoms (Cines et al. 2015). Patients with dementia may suppress their suffering by utilizing emotion-oriented coping mechanisms; however, depression and not awareness of memory deficits appears to be the key driver of quality of life (Boer et al. 2007). The first case of a PAD case involving a dementia patient reported in an annual report by the RTE (RTE Annual Reports 2004), involved opposing views, between the treating physician and the independent physician, regarding the patient’s condition. Interestingly, the two general practitioners differed in their perspectives regarding the suffering condition. While the treating physician argued that the patient was suffering unbearably due to his dependence on others, his loss of decorum and loss of self-respect, the independent physician, member of the Euthanasia in the Netherlands Support and Assessment Project (SCEN), argued that “the patient’s awareness of his shortcomings would decrease as his illness took its course [and] his suffering would become less unbearable over time” (RTE Annual Reports 2004). In this case, the independent physician assumed that the patient would eventually develop anosognosia and hence his suffering would become tolerable. This frame of thought is loosely based on the time a patient must endure for his awareness to deteriorate; this argument is debatable as many factors (e.g. vascular events, depression) can alter the time of onset of anosognosia which makes this clinical entity unpredictable. To this extent, the bioethical issue regarding the time a patient must endure to be categorized as having unbearable suffering becomes a topic of discussion. Bolt et al., suggest that doubt about whether suffering is unbearable might be a factor that explains why physicians find it less conceivable to grant a PAD request to patients with dementia, psychiatric disorders or who are tired of living (Bolt et al. 2015). Further complicating this landscape, the presence of anosognosia might incentivize the physician who is less likely to grant a PAD to opt for non-intervention or refuse to complete the AED, as they might interpret that the patient is no longer suffering unbearably. These same authors suggest that moral objections, not related to the criteria of due care, also influence the decision-making process of carrying out an AED in cases of advanced dementia (Bolt et al. 2015). A patient with AD who develops anosognosia no longer has a clear idea of his illness, the situation that he is in, the prognosis and his alternatives. In this situation, a physician evaluating this patient can no longer fulfill the due care criteria required to perform the PAD.

Advance directives should not only be prepared in an early stage of the AD but at this point, the attending physician should ascertain and document the patient’s decisional competence to guarantee that the request was voluntary. While the AED must be updated regularly, and an update is no longer appropriate once the patient develops anosognosia, an update is no longer necessary and is ethically inappropriate. The AED should allow the attending physician to recognize the opportune timing to perform the PAD; additionally, in the presence of anosognosia in patients with dementia, the input of the primary caregiver and family members should be incorporated early in the decision-making process.

### Perspectives from two societies

Only a rather small percentage of physicians (i.e. 7%) have ever performed a PAD in patients suffering from causes other than cancer and other severe physical diseases (i.e. early-stage and advanced dementia, psychiatric disorders, and patients who is tired of living) in a survey among 2269 Dutch general practitioners, elderly care physicians and clinical specialists (Bolt et al. 2015). While most physicians in the Netherlands would grant a PAD request for cancer (85%) or other physical diseases (82%), less than half would grant a PAD request for early-stage dementia (40%), psychiatric diseases (34%), advanced dementia (29–33%), and patients tired of living (27%) (Boer et al. 2007). The three most commonly referred reasons for requesting PAD are, pointless suffering, loss of dignity, and weakness (Jansen-van der Weide et al. 2005). Physicians have the right to refuse a PAD request; nonetheless, several cases where a physician had refused to grant a PAD have been reported and questioned by the media (Bolt et al. 2015). Among the reasons commonly associated with refused requests are, being tired of living, being a burden to their family, and depression (Jansen-van der Weide et al. 2005). Furthermore, regional differences exist in the Netherlands but are more pronounced in Belgium. Although the province of North Holland, which includes Amsterdam, had the highest prevalence of PAD in the Netherlands, these regional differences could not be explained by demographic, socioeconomic, or health-related differences; consequently, more detailed research is needed to specify how and why the practice of PAD differs between regions in the Netherlands (Koopman and Putter 2016). Meanwhile, keeping in mind that underreporting might be an issue, regional differences in Belgium are more pronounced, as the Dutch-speaking region of Flanders has higher PAD reporting rates (i.e. 73% to 58%). Flemish physicians had a more positive attitude toward PAD (i.e. 92% to 87% acceptance to the administration of euthanasics and 82% to 65% acceptance toward euthanasia as part of a good end-of-life care) and granted more PAD requests (i.e. 51% to 38%) compared to the French-speaking region of Wallonia (Cohen et al. 2012).

Although contrary to public opinion, physicians incline to support PAS over euthanasia; surveys in the United States, Europe, and Australia consistently show lower support for PAD among physicians than the general public (Emanuel et al. 2016). Furthermore, a survey conducted in seven countries reports approval of PAS ranging from 30% in France to 54% in the US (Emanuel et al. 2016); meanwhile, among Mexican physicians, 40% agree with PAD, 44% are against it and 16% are undecided (Lisker et al. 2008). Dutch physicians have a favorable opinion of PAD (i.e. 86% acceptance rate); similarly, physicians in Belgium approve of PAD in 81% (Emanuel et al. 2016). Conversely, only 10% to 33% of physicians in Finland and the Netherlands support PAD in advanced dementia, provided an AED exists (Tomlinson and Stott 2015). In the Netherlands, 23% to 58% of primary and secondary care nurses support PAD in patients with dementia (Tomlinson and Stott 2015). Public opinion, as well as the medical profession, are influenced by stigma generated by PAD in patients with dementia. Dementia patient caretakers holding religious views consistently report lower acceptance of PAD in dementia (Tomlinson and Stott 2015). An article analyzing the metaphors surrounding public and academic discourse regarding the euthanasia debate in patients with dementia states that this debate is morally loaded and has stigmatized AD, thus influencing people’s opinion toward legalization of euthanasia as an end-of-life alternative (Johnstone 2013). Public and academic discourse might be an influencing factor in how physicians view the term euthanasia and PAS. The psychological meaning of the term ‘euthanasia’ varies between medical students and physicians. At the beginning of their education, medical students in Mexico have ambivalent attitudes and posit a negative and positive psychological connotation on the term ‘euthanasia’; while advanced students and physicians have both positive attitudes and psychological meaning in regards to ‘euthanasia’ (Río and Marván 2011). Similar to the general population, religion skews the perspective physicians have toward PAD. Religious physicians in the Netherlands find it less conceivable that they would perform a PAD than non-religious physicians, rejecting PAD probably on the grounds of religious principles (Marwijk et al. 2007). Correspondingly, Mexican physicians who are against PAD procedures based their opinion mainly on moral and religious arguments (Lisker et al. 2008). Among medical students, their outlook concerning PAD varies depending on the type of medical school they attend, with students enrolled in lay institutions approving of PAD in 68% and students attending institutions with a religious orientation with a 33% approval rate (Loria et al. 2013).

#### A new perspective

A physician has the ethical responsibility to do no harm, *primum non nocere*. In regards to PAD in patients with dementia, this principle applies to the patient, the family or primary caretaker, and the physician. All three parties must be considered and included in the decision-making process of an AED. The practice guideline update for mild cognitive impairment (MCI), advocates for the physician to counsel patients and families about long-term projects including driving safety, finances, estate planning, and advance directives (Petersen et al. 2017); consequently, the discussion of an AED, among other long-term planning topics, should be addressed in early stages of AD. The attending physician must have careful consideration of the patient’s receptiveness and understanding of what MCI diagnosis and prognosis entails. It is the opinion of the authors that the discussion about sensitive topics such as AED should be initiated under the patient’s terms at the MCI stage, as evidence received by the RTEs reveals that patients and their relatives at end stages of life at times feel under great pressure to produce an AED (RTE Annual Reports 2004). This is where geriatric assent can facilitate this conversation, given that an AED should reflect the patient’s long-standing values and preferences, incorporate the patient's values and preferences; as well as guarantee the protection of the patient’s remaining autonomy.

The role of the family varies between countries and clinical settings. In the Netherlands, the family of the patients requesting PAD plays an important role in the decision process of granting the request, as their involvement and support influence end-of-life clinics’ decisions to grant the request (Snijdewind et al. 2015). In the United Kingdom caretakers of patients with dementia maintain that the decision for PAD should be autonomous; however, they consider that the decision-making process should be shared with the family (Tomlinson et al. 2015). The information provided by the family members or primary caretakers plays an important role in the RTE’s assessment whether the criteria of due diligence were met by the treating physician (Tomlinson et al. 2015). Family members decide to present the AED, undertaking great amounts of responsibility by initiating the deliberation process; furthermore, this responsibility often causes moral distress (Hertogh 2009). In the Netherlands, AED for euthanasia is infrequently adhered to in the case of people with advanced dementia, having a limited role in advance care planning and end-of-life care of people with advanced dementia (Boer et al. 2011). While in Belgium, Chambaere et al. report that the end-of-life decision is typically made without the patient’s input (i.e. only 1.2% to 6.7% of patients with dementia are involved in the decision making process in cases of euthanasia), involving only the physician and the long-term family caregivers in the decision-making process (Chambaere et al. 2015). Furthermore, the decision to hospitalize nursing home patients with dementia in Belgium follow the physician’s orders and not an AED (Houttekier et al. 2014). The patients’ preferences and values regarding the end-of-life, the needs of relatives are factors that must be considered in the shared decision-making process of establishing an AED for patients with dementia (Dees et al. 2013). In Latin America, the role of the family members and caretakers of the patient with dementia is of great importance to the end-of-life debate. With an aging population and an inadequate social health care system, the patient-care burden in Mexico is undertaken by the family of the dementia patient (Montes de Oca and Hebrero 2008). Overall, among Mexican families, the burden of care of a dementia patient lies upon one or two family members, primarily female members, which can ultimately lead to a caretaker burnout syndrome (Montes de Oca and Hebrero 2008). In the context of Mexican culture, family dynamics are important factors to be considered when making end-of-life decisions regarding patients with dementia. When the economic burden becomes an issue for the family members of the patient, geriatric assent becomes of paramount importance as the right balance between the patient’s wishes and best interest for the family must be taken into consideration. Ideally, a patient must express his end-of-life wishes when competent via an AED or written directive, making sure his family physician and family members are aware of these wishes.

#### Future perspectives in Mexico

Colombia is the only Latin American country where PAD is legal, yet only limited information has been reported regarding this end-of-life option. The topic of PAD in patients with dementia became recently a matter of public discussion in Mexico following the suicide of a 72-year-old man recently diagnosed with AD in January 2018 (Zamarrón 2018). The patient left a suicide note in which he explained that he had decided to take his life due to his lack of desire to live with AD and to the absence of a legal framework allowing for PAD in dementia. Mexico has made progress legislatively in regards to end-of-life care, as the country’s capital is expected to submit the final version of an amendment to its federal constitution within the next year, which could legalize PAD under the umbrella term of a dignified death (i.e. “Muerte Digna”). In Mexico 56.4% of the general population favour legalising PAS, and 58.3% favour legalising euthanasia; furthermore, 71.3% of the general public considers that changes in the law are necessary to allow patients to have a say in end-of-life decisions (Derecho and a Morir con Dignidad, 2016). If support for the right to a dignified death in Mexico City leads to PAD being legalized, then the institutions and end-of-life medical support network for adequate euthanasia and PAS application in Mexico should be established.

Mexico City could be inspired by the blueprint provided by the Dutch and Belgian end-of-life care experience from the last 15 years. The legal framework for PAD should be altered regarding special cases such as patients with dementia who request PAD. Elaborating and updating an AED should be part of the end-of-life management of patients requesting a PAD, serving as a mechanism to manage the cases where patients with AD develop anosognosia as their cognitive decline progresses. In addition to addressing special cases involving AEDs, the law *should* establish the institutions that regulate PAD. These institutions must provide specific education in PAD and palliative care measures for patients with dementia. Mexican physicians lack adequate training in palliative and end-of-life care (Human Rights Watch 2014); additionally, not enough funds in the health care system are allocated to address the end-of-life care needs for “all” patients. End-of-life care and more specifically death as a topic are not adequately included in the curriculum in medical schools in Mexico. Out of 160 medical schools in Mexico, only six provide a palliative care medicine course; while only two schools make such a course compulsory (Human Rights Watch 2014). During their medical school education, young physicians *should* be trained to have conversations with their patients regarding end-of-life care options and understand the importance of AEDs. Physicians must be upfront and honest with dementia patients regarding their functional prognosis while integrating clinical outcomes such anosognosia for the patient to adequately consider all end-of-life options and state any desired interventions in the patient’s end-of-life directive once they are no longer competent. Furthermore, clinical determination of competence is a skill needed by a physician to adequately assess end-of-life decisions.

An important aspect leading to the success of the PAD model in the Netherlands is the close patient-physician relationship. This close relationship between patient and physician changed due to the recent implementation of end-of-life clinics in the Netherlands (i.e. Levenseindekliniek). Shortly we should have more information about how these clinics not only address the needs of end-of-life care but impact the importance of a close patient-physician relationship in PAD. Mexico does not have a health care system where the primary physician has a lasting interaction with the patient. For a system resembling the Dutch PAD framework to satisfactorily work in Mexico, reforms in the health care system *must* take place. According to the latest national health care survey (“Encuesta Nacional de Salud y Nutrición”) in 2016, 13.4% of the Mexican population did not have any health care; meanwhile, 22.7% of respondents reported having paid for medical care (Secretaría de Salud 2017). Although no surveys have specifically reported the proportion of the population with a family physician, in actuality it is hard to envision patients attending follow-up consultations with the same physician. These patterns of primary medical attention suggest that the majority of the Mexican population does not have a family physician. The unsuitable patient-physician relationship generated by the Mexican health care system, the limited access to palliative care medicine, and considering that half of the population is poor or very poor, the need of education not only to health care providers but society as a whole, regarding end-of-life topics, becomes increasingly important.

## Conclusion

To the best of our knowledge, this is the first article that addresses the clinical considerations regarding PAD including determination of decisional competence and awareness of memory deficits (i.e. anosognosia). Furthermore, we contrast perspectives from culturally distinct societies as it pertains to the decision-making process in PAD for patients with dementia. Previous work regarding the role of AED in end-of-life decisions involving patients with dementia has revolved around the ethical relevance of shared understanding and reciprocity, thus creating a window of opportunity for shared decision-making (Hertogh 2009). Meanwhile, anosognosia has been proposed as a condition in the natural history of dementia that limits the applicability of AED, hence some have concluded that due to clinical and ethical reasons, AED could never lead to a PAD in patients with advanced dementia (Hertogh et al. 2007). Nonetheless, after 2012 an increase in euthanasia reporting has occurred, with a notable increase of PAD cases involving dementia patients. This upward trend in reporting is a reflection of the changing attitudes toward the management of PAD requests by attending physicians partially resulting from the work of the regional committees to educate the medical profession, the role of end-of-life clinics, and overall increased awareness by the population. This work serves as an update of the recent changes regarding the outlook of end-of-life perspectives toward PAD and AED in patients with dementia.

Countries that are on track to legalize PAD should be stimulated to use the knowledge attained through the 15 years of experience obtained through the Belgian and Dutch model while incorporating suitable aspects from common law jurisdictions (i.e. United States of America and Canada) that are relevant to their social and cultural context. In this article, the authors provide the social and cultural context that must be considered when implementing new end-of-life legislation. The example of Mexico City’s new constitution will pose new challenges to the health care system and society as a whole if PAD is legalized. New institutions or committees within current institutions in the health care system must be established to regulate the practice of PAD similarly to the Dutch and Belgian model. The topic of end-of-life care including complex conditions such as dementia must be introduced not only into the legislative, medical and academic discourse but also into the public domain, as the social impact of these legislative changes can lead to erroneous interpretations. All in all, the new law will empower patients with the right to choose what and how much suffering they are willing to bear, as well as when and how this suffering will be handled.

This work attempts to provide a perspective on a clinical approach that engages with conditions (i.e. anosognosia) that alter the clinical presentation in patients ranging from cognitive complaints to overt dementia. A discussion about the clinical approach for PAD in AD was elaborated in the context of two different legal but inherently similar bioethical frameworks. Assessment of awareness of memory deficits allows the clinician to evaluate comprehensively the course of the patient’s cognitive decline. Anosognosia could potentially be a clinical condition that deters PAD action. The patient’s loss of competence represents the breaking point for the decision-making process concerning PAD in patients with dementia. PAD is not the moment a life is finished, it is a well-thought-out process where the patient and the physician together decide that the struggle against this disease must end. Overall, the formal request for an AED marks the beginning to this end. The end of a journey poised on the patient-physician bond. A physician might not always be capable of healing but should always be capable of comforting. It is an honor for a physician to be entrusted with the assistance of birth, the maintenance of health and the observance and assistance to provide death with dignity.

## Electronic supplementary material

Below is the link to the electronic supplementary material.

Supplementary file1 (DOCX 62 kb)

## Abbreviations

AD: Alzheimer’s disease
AED: Advance euthanasia directive
PAD: Physician-assisted death
PAS: Physician-assisted suicide
RTE: Regional Euthanasia Review Committee

## Footnotes

1 Decisiones médicas sobre el final de la vida en pacientes con enfermedad de Alzheimer. Un abordaje interdisciplinario: Fontamara (2017), p. 4.

2 For an in-depth take on the neural correlates of disorders of consciousness we refer the reader to the text by Di Perri et al. (2014) and Mondragon et al. (2019) for anosognosia in AD.
