# Supplementary material for: Bioethical implications of end-of-life decision-making in patients with dementia: a tale of two societies
Source: Monash Bioeth Rev. 2020 Apr 25;38(1):49–67. doi: 10.1007/s40592-020-00112-2 (PMC7205770; doi:10.1007/s40592-020-00112-2)
Supplement: Supplementary file 1 — Supplementary file1 (DOCX 62 kb) [file 40592_2020_112_MOESM1_ESM.docx]

1. **Abridged legal framework of physician-assisted death**

In the Netherlands, PAD was legalized in 2002 under the Dutch Termination of Life on Request and Assisted Suicide Act, with the aim of providing a legal framework and increase the degree of due care exercised by physicians when performing a PAD [25]. An attending physician is a medical doctor who has a medical treatment contract with the patient [5]. The statutory procedure governing PAD assumes that the attending physician terminates the life of the patient. Under Article 446 of the Dutch Civil Code, a medical treatment contract exists if, “in the pursuance of a medical occupation or enterprise, a natural or legal person undertakes to carry out medical interventions that directly affect the patient” [5]. Under Dutch law (i.e. Section 7(2) of the Burial and Cremation Act), the physician must submit a written report of the termination of life, with reasons and observance of due care requirements, in communication with the municipal forensic pathologist after performing the act [5, 25]. The pathologist then performs an external examination of the body and verifies the procedure and substances used to terminate the patient’s life [5]. This report is forwarded to a regional committee (i.e. a lawyer, a physician and an expert on ethical or philosophical issues), which determines within six weeks if statutory due care requirements were met [5,25]. If the physician has complied with all due care requirements, the procedure is deemed complete; however, if lack of due care is determined, the case is forwarded to the Public Prosecution Service and the Health Care Inspectorate [5,25,44]. All cases suspected of having not complied with the due care criteria must be discussed with all five regional RTEs before a final determination is made [5]. While termination of life has not been legalized, if the conditions for due care are met and the termination is carried out by a physician, the PAD is not considered to be punishable [25].

In 2002, euthanasia was legalized in Belgium, while PAS is not explicitly permitted [63,64]. No age restrictions are imposed by Belgian law (i.e. 2014 Child Euthanasia Amendment) [63]. According to the Belgian Euthanasia Act of 2002, the patient must repeatedly state his desire for PAD; additionally, the physician must ascertain that the requests are voluntary, well-considered, and not the result of coercion [19]. The request must be written, dated and signed by the patient; not be older than five years and must be witnessed by two individuals [14]. The Belgian Euthanasia Act stipulates that in cases involving non-terminal illnesses (e.g. quadriplegia and neurodegenerative disorders) a one-month moratorium on the PAD must elapse [19]. Belgian law dictates that the physician must report the PAD to the Federal Control and Evaluation Commission [63].

Belgian law does not explicitly prohibit PAD on patients with a diagnosed disease leading to (to be looked at) dementia. Alzheimer’s disease (AD) is a progressive cerebral disease defined by a clinical and a pathological component. Clinically, this disease involves anterograde memory impairment and deficit in one or several of the following cognitive domains: language, visuospatial ability, praxis and executive functioning [65]. Patients with dementia may request PAD under two conditions. First, while preserving their decision-making capacity at an early-stage, the patient is experiencing extreme mental suffering [1]. Secondly, involving “late-stage [dementia] patients who previously completed an advance euthanasia directive (AED) requesting death when their symptoms become severe” [1]. The RTE expects the physician to act “very cautiously” and to have “extremely careful decision-making” when a patient suffering from dementia requests euthanasia [25]. The legal framework around the world has extensively been discussed in the medical law literature [3, 63] and is an evolving topic in developing countries (i.e. countries with medium and low inequality-adjusted human development index) [7]. Considering that patients with dementia are recognized to have prolonged survival than terminal illnesses, a third physician (i.e. expert on the disease, according to the law) must be consulted when request before the request for PAD is granted by the treating physician [20]. Nonetheless, euthanasia for patients with dementia is currently a topic of debate in Belgium due to its historical and political intricacy [64], moral implications [19], and the complexity associated with the physician’s end-of-life decision-making [56].

1. **Opposition to PAD and AED in patients with dementia**

Recently, an investigation was launched by Dutch public prosecutors, regarding a PAD of an advanced dementia patient who had previously signed an AED [66]. The RTE concluded that the geriatrician “did not act in accordance with the criteria of due diligence” required by law and labeled the AED as “ambiguous and contradictory” [66]. Interestingly, as the patient deteriorated and asked if she wanted to proceed with the PAD as she had previously requested, she refused; the case is complex because she showed signs that she no longer understood what the euthanasia procedure entailed [66]. The case has garnered notoriety, as the topic of PAD for non-competent individuals has become public discourse among physicians. In early 2017, a group of 33 physicians led by psychiatrist Dr. B. Chabot signed a manifesto urging the medical field to stop the normalization of “defenseless people” [66]. The position of the Dutch Medical Association regarding the current attitude of physicians to PAD for advanced dementia cases is that although technically legal, physicians have “strong moral objection” if adequate communication is no longer possible [66]. The notoriety of this case has brought forward the pending issue regarding the appropriate measures that physicians must consider to avoid such an outcome. This case exemplifies that complying with the due care criteria and notification procedure is only part of the PAD in dementia discourse; furthermore, an appropriate clinical approach for PAD in dementia is necessary. Attending physicians must exercise a high degree of caution in cases involving PAD requests from dementia patients. Regarding cases of patients with dementia requesting PAD, the RTE recommends the physician to be “extremely cautious” and the assessment process should involve “extra due care” [25].

Opponents of PAD in patients with early dementia argue that although competent to make a decision, at this stage of the disease they can still have months (or years) of quality life [19]. Nonetheless, since PAD is considered wrong in later stages of dementia as this contravenes competency and autonomy considerations [19]; thus leading to a bioethical quandary regarding the temporality of the PAD decision-making process. A good patient-physician relationship is of paramount importance in the PAD shared decision process [6,54]. The decision process preceding a PAD request can be paramount for a physician. Three phases have been identified associated with the physician’s emotions in regards to the PAD decision-making and implementation process. The primary care physician experiences tension before the PAD event, followed by loss during and relief after the event [27]. Overall, it is understandable why a request for PAD can be distressing to the physician. Among the factors associated to performing a PAD are an established patient-physician relationship, the feeling of loneliness after performing the procedure, the role of the family of the patient, and pressure from society regarding the responsibility and end-of-life procedure [27]. Opponents of the AED as a valid document during the end stages of dementia contend that ethically the execution of a directive for these patients is unfeasible since the communication process between the patient and the attending physician is disrupted, thus shared understanding is impossible [41, 67]. Application of AEDs in patients with advanced dementia seems limited based on the opinion of physicians. Physicians seem reluctant to replace adequate verbal communication with an AED, as verbal communication allows the verification of the voluntariness of patient’s request and the condition of unbearable suffering [68].

End-of-life decisions have become more prominent in Dutch society. Before a PAD becomes a viable option, good palliative care must be provided; in the end-of-life decision process, PAD should be the last option. RTEs are not competent to assess end-of-life cases where continuous deep sedation was the cause of death since this is considered a normal medical procedure [5]; furthermore, deep sedation as an end-of-life decision is at the moment also under discussion. Physicians have different approaches when conducting end-of-life decisions regarding cancer and dementia patients. In Belgium, dementia patients are more likely to have treatment withheld or withdrawn (OR 1.40, 95% CI= 1.00-1.96, p= 0.048) than cancer patients and consulted the caregivers more often about end-of-life decisions (OR 1.99, 95% CI= 1.19-3.33, p= 0.009) [56]; additionally, 19.5% of nursing home residents with dementia in the region of Flanders were hospitalized in the last month of life with 37% of the request for hospitalization coming from relatives [57]. While in the Netherlands, 42.3% of physicians withheld life-prolonging treatment and withdrew treatment in 53.7% of patients with dementia in a nursing home setting who rarely had an AED (i.e. 4.9%) [69]. In the Netherlands patients and physicians are increasingly opting for continuous deep sedation as an end-of-life alternative; showing increases from 8.2% in 2005 to 12.3% in 2010 reaching its highest rate in 2015 of 18.3% [70]. Meanwhile, in Belgium, this trend is more variable since continuous deep sedation was chosen as an end-of-life option in 8.2% of the cases in 2001 increased to 14.5% in 2007, and declined to 12.0% in 2013 [71].

1. **Current perspectives of PAD and dementia in Mexico**

The World Health Organization (WHO) recognizes dementia as a public health priority in middle-income countries such as Mexico [72]. Although in 2014 only 86,000 people had a dementia diagnosis in Mexico, by 2050 this number is expected to rise to over 3.5 million [73]. This demographic shift represents a challenge to the health care system due to expected high costs, as hospitalizations are a costly burden to a health care system. A large portion of dementia patients in Latin America spends their last days hospitalized: Peru 83.3% (rural population); Cuba 41.4%; Dominican Republic 29.1%; Peru 31.0% (urban population); and Mexico 31.3% [74,75]. A debate regarding PAD in patients with dementia must be initiated, not only to address the bioethical concerns regarding individuals but also to address end-of-life care as a public health topic. To the authors’ knowledge, no academic text has delved into the subject of PAD in patients with dementia in Mexico or Latin America.

In Mexico, AD has a prevalence of 7.3% for people over the age of 60 and an annual incidence of 27.3 per 1,000 persons [73,76]. In Latin America, AD prevalence is 8% and is expected to quadruple between 2015 and 2050 [73,74]. Sixty percent of people with AD are expected to live in developing countries by 2020 [77]. AD risk factors in the Mexican population are similar to those of other Latin American and developing countries, including female sex, metabolic syndrome, cardiovascular disease, low education, socioeconomic inequality, and depression [73,74]. Recent population projections estimate that about 3.5 million geriatric patients will be diagnosed with AD in Mexico in 2050; furthermore, the number of people fully dependent on others will increase from 349 to 613 million in the next 30 years worldwide [73]. Accordingly, the main cause of dependence around the world is dementia. Dementia contributes to 12% of years lived with dependence, higher than cardiovascular diseases (9%), musculoskeletal diseases (8%), and oncological diseases (4%) [73]. Emerging economies are set to absorb the greatest economic burden, as the portion of dependent patients is greater than in countries with a high or a very high inequality-adjusted human development index. Among the countries that will confront this challenge in the next decades are Mexico, Cuba, Dominican Republic, and Peru [78].

During the 2013 Group of Eight meeting, world leaders made a call to the international community to step up to the challenge posed by dementia. Among the domains to be addressed were prevention, quality of life, early diagnosis, treatment, research, and social acceptance; nonetheless, end-of-life care was not an emphasized domain. The international community must also focus on end-of-life care for dementia patients in order to guarantee an adequate quality of life and social acceptance in this growing population. Similar to the G8 directives, the 2015 Mexican National Plan regarding AD also addresses the domains focused by the G8, thus omitting end-of-life care as a topic of interest [73].

1. **Palliative care in Mexico**

The perspective from a palliative care point of view must also be part of the discourse involving end-of-life care. In order to exercise the right over end-of-life decisions, a patient must have acquired information and access to palliative care. Regarding patients with dementia, adequate palliative care measures must be guaranteed in order to ascertain that the PAD request is not coerced by the inadequate end-of-life care. In 2014, Human Rights Watch reported that in Mexico hundreds of thousands of people do not have access to palliative care; specifically, five out of the 32 states, amounting to a population of 7.5 million, do not have public access to palliative care [60]. Meanwhile, five other states have palliative care services in their capital and only five states provide palliative care in various cities [60]. Along with regionally centralized palliative care, deficient access to palliative care is inexistent in small cities or rural areas. According to the Organization for Economic Co-operation and Development (OECD), Mexico has among the poorest quality of death indices, ranking 43rd out of 80 in the world and 11^th^ out of 17 in Latin America, with a quality of death index for palliative care of 42.3 (i.e. as a point of reference, the United Kingdom ranks the highest with an index score of 93.9, Belgium ranks fifth with an index of 84.5, and the Netherlands ranks eight with an index score of 80.9) [79]. The same reasons, in addition to poverty and an indigenous population with poor access to medical technology, that affect patient access to PAD explain the deficient access to palliative care in Mexico. First, the development of health care human resources competent for addressing end-of-life topics is deficient. Very little or nothing at all is discussed during the formative years in medical schools, nursing schools, clinical psychology, and social work departments; additionally, few postgraduate programs incorporate these topics to their training plan [60]. A health care worker, who is not trained to attend end-of-life care needs, is incapable of providing adequate care for terminally ill patients. Although few specialists have a formal training to provide palliative care in Mexico, there is a need for guidelines to be implemented in palliative care units [60]. In addition to the insufficient development of human resources and accessibility problems regarding palliative care, very few insurance policies cover palliative care costs [60]. Access to palliative care is a right, established by the General Health Law in Mexico, available for all patients with less than six months of life expectancy [9]. The new constitution of Mexico City is expected to include legislation regarding end-of-life topics. However, the exercise of this right will be compromised without adequate access to palliative care; correspondingly, patients with dementia are to be considered a vulnerable group.

Currently, in Mexico, the only three available options for end-of-life care are, palliative care measures, measures established by an advance health care directive, and withdrawing or withholding treatment [9]. Mexican physicians must know the legal framework and end-of-life options currently available; thus allowing an adequate counseling of dementia patients and their family members. Family physicians should explain the different clinical outcomes associated with the progression of cognitive decline (e.g. anosognosia) and assess decisional competence clinically. Euthanasia is not the precise act of ending a life; it is the result of a joint decision between the attending physician and the patient that the battle against a disease has ended. PAD is a shared decision that epitomizes the importance of the patient-physician relationship. It is an alternative for the final phase of life to avoid excessive suffering; an option circumscribed by established "due care criteria", which protect the patient, the physician, and society from unregulated end-of-life actions. The last ten years cases involving patients with dementia requesting PAD has become a relevant topic of discussion among the general population, clinicians, and in academia. Arguments in favor of PAD as an end-of-life option for patients with dementia emphasize that these end-of-life options empower the patient and the physician because these alternatives preserve autonomy until the end of life; thus preserving human dignity and preventing unnecessary humiliation. However, opponents of PAD in patients with dementia argue that euthanasia in this population along with other neuropsychiatric disorders can lead to a slippery slope; substantiating that palliative measures are sufficient to provide care for dementia patients’ needs at the end of their lives. To think that the end-of-life decisions can be reduced to only providing palliative measures is to dehumanize the patient since a physician must always consider the physical, mental and social well-being of the patient. Medically assisted death is not the antithesis, strictly dialectical meaning, of palliative care, but an alternative to it. Both are valid end-of-life options, which are the resulting synthesis between "overtreatment" (i.e. therapeutic obstinacy or dysthanasia) and the natural history of the disease.

**References**

1. Dyer O, White C, García Rada A. (2015). Assisted dying: law and practice around the world*. BMJ. 351,* h4481.
2. Saad TC. (2017). Euthanasia in Belgium: legal, historical and political review. Issues *Law Med. 32(2*), 183-204.
3. Boughey JGF, Graff-Radford NR. (2007). Alzheimer's disease. In: Schapira A, (1st ed). Neurology and clinical neuroscience, Philadelphia: Mosby Elsevier; 846-58.
4. Sheldon T. (2017). Dutch geriatrician faces charges over euthanasia case. *BMJ. 359*, j4639.
5. van Delden JJ. (2004). The unfeasibility of requests for euthanasia in advance directives. *J Med Ethics. 30(5)*, 447-51.
6. Kouwenhoven PS, Raijmakers NJ, van Delden JJ, et al. (2015). Opinions about euthanasia and advanced dementia: a qualitative study among Dutch physicians and members of the general public. *BMC Med Ethics.* 16,7.
7. Hendriks SA, Smalbrugge M1, Deliens L, et al. (2017). End-of-life treatment decisions in nursing home residents dying with dementia in the Netherlands. Int J Geriatr Psychiatry. 32(12), e43-e49.
8. van der Heide A, van Delden JJM, Onwuteaka-Philipsen BD. (2017). End-of-Life Decisions in the Netherlands over 25 Years. N Engl J Med. 377(5), 492-494.
9. Chambaere K, Vander Stichele R, Mortier F, et al. (2015). Recent trends in euthanasia and other end-of-life practices in Belgium. N Engl J Med. 372(12), 1179-81.
10. World Health Organization. (2012). Dementia: A Public Health Priority. Washington, DC. Retrieved from http://apps.who.int/iris/bitstream/10665/98377/1/9789275318256_spa.pdf [Accessed February 2018].
11. Gutiérrez-Robledo LM, Arrieta-Cruz I. (2015). Dementia in Mexico: The need for a National Alzheimer´s Plan. Gac Med Mex. 151(5), 667-73.
12. Parra MA, Baez S, Allegri R, et al. (2018). Dementia in Latin America: Assessing the present and envisioning the future. *Neurology. 90(5)*, 222-231.
13. Prince M, Comas-Herrera A, Knapp M, et al. (2016). World Alzheimer Report 2016. Improving Healthcare for People Living With Dementia: Coverage, Quality and Costs Now and in the Future. Alzheimer's Disease International; retrieved from https://www.alz.co.uk/research/world-report-2016 [Accessed February 2018].
14. Llibre Rodriguez JJ, Ferri CP, Acosta D, et al. (2008). Prevalence of dementia in Latin America, India, and China: a population-based cross-sectional survey. Lancet. 372(9637), 464-74.
15. Ferri CP, Prince M, Brayne C, et al. (2005). Global prevalence of dementia: a Delphi consensus study. Lancet. 366(9503), 2112-7.
16. Sousa RM, Ferri CP, Acosta D, et al. (2009). Contribution of chronic diseases to disability in elderly people in countries with low and middle incomes: a 10/66 Dementia Research Group population-based survey. Lancet. 374(9704), 1821-30.
17. The Economist Intelligent Unit. The 2015 Quality of Death Index Ranking palliative care across the world. Retrieved from https://www.eiuperspectives.economist.com/sites/default/files/2015%20EIU%20Quality% 20of%20Death%20Index%20Oct%2029%20FINAL.pdf. [Accessed February 2018].
18. **Regional Euthanasia Review Committee Annual Report cases involving dementia patients**

**Case 2004-3:** **Alzheimer’s patient**

*A 65-year-old man had been suffering from Alzheimer’s disease for the past three years. The depression he suffered as a result of his illness could be treated successfully with medication. The patient also went to a nursing home for treatment during the day. The fact that he could no longer function independently in any way was a source of unbearable suffering. He also suffered from his understanding of what the future held for him, as the dementia progressed. When he was first diagnosed with Alzheimer’s, he told his attending physician that he had no wish to see it through to the bitter end. In the year before euthanasia was performed, he repeatedly asked his doctor to help him commit suicide.*

*The attending physician consulted an independent physician, who agreed that the patient was suffering unbearably from his dependence on others, his awareness that he was deteriorating and losing all sense of decorum, his loss of independence and self-respect, and the knowledge that his situation could only get worse.*

*All the same, the independent physician could not entirely empathise with the patient. He also felt that since the patient’s awareness of his shortcomings would decrease as his illness took its course, his suffering would become less unbearable over time.*

*According to the independent physician, though the patient had repeatedly requested euthanasia over a longer period of time, his ability to make an informed decision was debatable, given his inability to follow the physician’s reasoning during their interview. The independent physician therefore concluded that the due care criteria had not been fulfilled.*

*After receiving the independent physician’s assessment, the attending physician consulted three specialists: a psychologist, a nursing home doctor and a geriatric psychiatrist. From the examination that they each carried out independently, these specialists came to the conclusion that the patient was not suffering from depression, wanted to maintain control over his life and was aware that his illness would eventually deprive him of it. They each came separately to the conclusion that the patient was capable of making a voluntary, well-considered request for euthanasia, and that he was aware of the implications of his choice. Given the conclusion reached by these three specialists, the attending physician decided to grant the patient’s request. The patient died as a result of assisted suicide.*

*In its assessment, the committee considered the fact that though the independent physician had questioned the ability of the patient to make an informed decision, each of the specialists consulted later came independently to the conclusion that he was most certainly able to do so, and was quite capable of deciding what he wanted and why. According to the committee, the attending physician had fulfilled the criteria of consulting at least one other physician. Confronted with the conflicting opinions of the independent physician and the three specialists consulted later, and given his own views in the matter, the attending physician rightly accorded more significance to the opinion of the three specialists. According to the committee, the attending physician could decide on that basis to assist the patient to commit suicide.*

*The committee found that the physician had acted in accordance with the due care criteria.*

**Case 2005-4: Voluntary and well-considered request – dementia**

**The attending physician called in a geriatric psychiatrist to determine whether the patient was capable of informed consent. The committee examined whether the request was voluntary and well-considered and whether the patient’s suffering was unbearable, with no prospect of improvement. The due care criteria were fulfilled.**

*In June 2003 the patient, an 83-year-old woman, was diagnosed with Lewy body dementia, which is incurable. She was treated with Exelon and showed a good cognitive response. At the same time she was given medication for her hypokinetic rigid syndrome and her sleeping problems. Her motor symptoms became worse and she continued to be restless at night.*

*The patient was suffering from the fact that she could no longer function independently and had become ADL-dependent, that she was losing control of her body and hence was incontinent and could no longer walk properly, and that her cognitive functions were impaired so that she no longer had full control of her mind, especially at night. It was vital to the patient to be in control of her mental faculties. She knew that she was suffering from a progressive condition (Lewy body dementia) and was bound to deteriorate. She realised that she would eventually no longer be able to make her wish for euthanasia clear and that she would lose her autonomy and be admitted to a nursing home. The idea of consciously experiencing this unavoidable physical and mental decline, together with the fear of increasing loss of control over her mental faculties, was unbearable to her. Apart from the palliative measures already taken, there was no way to alleviate her suffering.*

*The patient first specifically requested euthanasia in July 2005, and repeated the request on a number of occasions thereafter. Two independent physicians were called in. The first was a psychiatrist and the second a SCEN general practitioner. They saw the patient in July 2005 and late September 2005 respectively. According to the report by the first independent physician, the patient described how she had noticed in the previous two-and-a-half years that her memory was failing and that she was finding it more difficult to speak and do sums. She had subsequently been diagnosed with Lewy body dementia. Since taking Exelon she had regained some control over her mind. She also said that she had difficulty in walking and was incontinent, which was a particular problem at night. She said that she became increasingly confused in the early evenings and usually slept badly at night. She then often felt frightened and despondent. In the afternoons she felt better and was still capable of enjoying things. The patient perceived her dependence as humiliating. She was finding life increasingly difficult, and she did not want to deteriorate any further. She would prefer to decide for herself when to die. She did not feel it was fair, especially to those around her, to take her own life or hasten death by refusing food and fluids. She had been a member of the Dutch Association for Voluntary Euthanasia (NVVE) for twenty years and had discussed her wish for euthanasia with her husband and five children. They found her decision hard to accept, but respected her choice.*

*According to the first independent physician, the patient’s cognitive functions were reasonably good at the time when he saw her. It was evident that she was used to applying her powers of reasoning, but also that she had to concentrate very hard. The physician stated that the patient’s depression and anxiety were appropriate in the circumstances. She had made her request for euthanasia voluntarily. Her ability to decide about this had not been influenced by her dementia or by depression. She was capable of informed consent, and her request was well-considered. Given the prospect that her symptoms would get worse and that she would eventually require nursing care, which she definitely did not want, there was no prospect of alleviating her suffering. The realisation that she was becoming more and more dependent on others and that she had less and less control over her body was unbearable to her.*

*The report by the second independent physician confirmed the patient’s case history and the fact that in July 2005 she had been examined by a geriatric psychiatrist, who had found her capable of informed consent regarding her request for euthanasia. The second physician reported that the patient walked unsteadily and could scarcely move her neck. After half an hour she clearly became fatigued and unable to concentrate. She felt she was suffering unbearably because she was powerless to control her mind and because her body was so feeble. She felt unable to decide things for herself, and knew that this would get worse as her disease progressed. She perceived her loss of ADL function owing to the rigidity of her muscles as humiliating. She also suffered from night-time restlessness and fears, as well as incontinence. As a result of all this she slept badly and was dependent on her husband. She suffered from the knowledge that her disease would make her cognitive and physical symptoms worse. She preferred euthanasia because she would not be able to commit suicide with modern sleeping tablets and she considered it undignified to hasten death by refusing food and fluids.*

*The second physician concluded that this was a voluntary, well-considered request for euthanasia by a patient who was fully informed about the nature of her disease. She was suffering unbearably, with no prospect of improvement and there was no other reasonable alternative. She had drawn up a euthanasia directive. He found that the due care criteria had been fulfilled, provided that the procedure for the termination of life was performed carefully. Euthanasia was performed in October 2005 with due medical care and attention.*

*In assessing whether the attending physician could be satisfied that the patient had made a voluntary and well-considered request, the committee considered the following factors. In her contacts with the patient, the physician had been aware of the patient’s views on euthanasia from the very outset. The patient had regularly expressed her wish for euthanasia as the disease progressed. To rule out the possibility of depression and to determine whether the patient was capable of informed consent regarding her request for euthanasia, the physician had called in a geriatric psychiatrist, who had concluded that the patient was not depressed and was capable of informed consent. The SCEN physician had also concluded on the basis of his interview with the patient that her decision had been voluntary and well-considered. In view of these facts and circumstances, the committee found that the attending physician could be satisfied that the patient had made a voluntary, well-considered request.*

*In assessing whether the physician could be satisfied that the patient was suffering unbearably, with no prospect of improvement, the committee considered the following factors. The letters from the specialists stated that the patient was suffering from progressive cognitive disorders accompanied by parkinsonism and hallucinations, in all likelihood caused by Lewy body dementia. This condition is incurable and patients deteriorate progressively. The patient was therefore suffering with no prospect of improvement.*

*As to whether the patient’s suffering was unbearable, the committee stated that she was suffering from her ADL-dependence and her loss of both physical and cognitive functions.*

*For a woman who had always been used to running her own life and being in control of her body, this physical decline was unbearable. She could not bear the thought that in a later stage of her disease she would require nursing care and would lose her autonomy. Her memories of the final stages of her parents’ and parents-in-law’s lives played a major part in this. She was also aware of what she would inevitably have to go through before very long. In view of all this, the committee found that the physician could reasonably have concluded that this particular patient was suffering unbearably. The physician had given the patient sufficient information about her situation and prognosis. The committee found that the physician had acted in accordance with the due care criteria.*

**Case 2006-3: Voluntary and well-considered request- dementia**

**This description of a case involving termination of a dementia patient’s life on request focuses on the request.**

*The patient, a woman between 70 and 80 years of age, had been diagnosed with Alzheimer’s disease. Further examination at a later stage revealed incipient dementia. Treatment was no longer possible. The patient knew exactly what her prognosis was. She had a full understanding of the disease and was well aware that no treatment would slow down or halt its progress. She had always been very independent, enterprising and lively, even after retiring. She had had first-hand experience of close relatives suffering from the same disease. Her own deterioration had become more and more evident, and her wish to die at a moment of her own choosing had become increasingly urgent. The physician had consulted an independent physician, who had seen the patient twice and given his written opinion that the due care criteria had been fulfilled. The assisted suicide had been carried out with due medical care and attention.*

*In early 2006 the patient had asked the physician to terminate her life at an early stage. She had subsequently repeated the request whenever she saw him. Although she had recently lost control of things in her day-to-day life, in conversations she always proved capable of keeping to a line of argument and referring back to what had been said earlier. When talking about termination of life, she was still able to approach the issue from various angles. She repeatedly stated that she was deteriorating rapidly, which the physician had also concluded. Years before, when Alzheimer’s disease had been diagnosed in her family, she had expressed her concern about this and had indicated her wish for euthanasia if ever the same fate should overtake her. She had drawn up advance directives. She said she was very much afraid that at some point her request that her life be terminated would no longer be granted. At first the physician had been uncomfortable with the point at which the patient had said during one of their conversations, that she wanted her life to be terminated, for at that stage he felt she was mentally “too well”. Gradually, however, he had become convinced that her time had come, given that her present situation and her prognosis were causing her unbearable suffering. The geriatric psychiatrist who had examined her and had diagnosed Alzheimer’s disease could, from a professional point of view, understand her wish not to wait for the next stage of the disease, and respected her wish for euthanasia. The attending psychiatrist had stated that initially, in January 2005, she appeared to be suffering from depression because her partner had died. However, the psychiatrist concluded that her cognitive disorders, especially her forgetfulness and her increasingly unbearable sense of despair (she was still clearly aware of her disease), were the key factors.*

*Given all the facts and circumstances of the case, the committee found that the physician could be satisfied that the patient’s request was voluntary and well-considered. When talking to her, he had always been alert to the degree of her suffering and the persistence of her wish. The physician stated that she had always been aware of the implications of her request, her situation and her prognosis. The geriatric psychiatrist, the attending psychiatrist and the independent physician considered that the patient was capable of informed consent and that she was very well aware of her situation and her prognosis.*

*The committee therefore found that the physician had acted in accordance with the due care criteria.*

**Case 2006-4: Unbearable suffering with no prospect of improvement- dementia**

**The facts of this case are the same as in Case 3, which concerns the same notification. The elements that mainly related to the patient’s suffering are discussed below.**

*The patient had always been very independent, enterprising and lively, even after retiring. She had had first-hand experience of close relatives suffering from Alzheimer’s disease. Her own deterioration had become increasingly evident. Mental stimulation had always been important to her. She had always been an extremely well-read person with a wide range of interests, but newspapers were now left unread and it took her months to get through a book. Her mind was growing dull. At first this made her feel that life had lost all its lustre, but as time passed she increasingly began to feel she was losing her identity. At the same time, she had less and less control over the organisation of her day-to-day life. She was losing her bearings, particularly her sense of time, and becoming dependent on help. She was aware that she had lost a large number of skills. She did not want to find herself unable to make her own decisions and totally dependent on others. She repeatedly expressed her despair at the idea of having to experience a slow, progressive intellectual decline. The idea that she would eventually be completely “useless” and would end up in a mental vacuum was unbearable to her. The realisation that she would no longer be able to make her own decisions was unacceptable to her.*

*Given all the facts and circumstances of the case, the committee found that the physician could be satisfied that the patient was suffering unbearably with no prospect of improvement. In making this assessment, the committee took account of the fact that the patient had been diagnosed with Alzheimer’s disease, which is incurable and leads to progressive deterioration.*

*As regards the unbearable nature of her suffering, the committee noted that the reports by the physician and the independent physician had indicated that she had always been very independent, enterprising and lively. Mental stimulation had always been important to her. She lived by her intellect and based her identity on this. Her progressive mental decline, her increasing dependence, and the fact that she was totally aware of this and had a clear idea of how the disease would progress were causing her to suffer. Her only prospect was further deterioration. The idea that she would no longer be able to make her own decisions and would be totally dependent on others was already unacceptable to her. The physician, as well as the independent physician, other physicians who had been consulted and friends of the patient’s, felt that under the circumstances her present suffering was palpably unbearable for a woman who had always been so mentally and physically active and enterprising.*

*Given all the facts and circumstances of the case, the committee found that the physician had acted in accordance with the due care criteria.*

**Case 2007-2: Voluntary and well-considered request- dementia**

**In the following case, the patient made her request for termination of life when she was in the incipient stages of dementia. The case shows how the physician enabled the patient to keep control of how her life would end. By consulting various experts in good time, he was able to satisfy himself that the patient had made a voluntary and well-considered request.**

*In autumn 2006, after two years of worsening symptoms such as noticeable slowness of mind and difficulty with basic arithmetic, the patient, a woman aged between 70 and 80 years of age, had been diagnosed with incipient Alzheimer’s disease. Her awareness of her disease and prognosis caused her great suffering, as did her growing dependence on others, her decreasing ability to communicate and make social contact, and her resulting isolation. She was losing control of her life. This suffering was unbearable to her.*

*Three months after the diagnosis, she had told the physician that she wanted euthanasia when the time came. She wanted to keep control over how her life would end. A few weeks before she died, she had specifically asked the physician to terminate her life.*

*Because the patient’s initial memory problems some years earlier had made the physician suspect she might be suffering from depression, and in order to respect her wish to keep control of her life, the physician had asked a geriatric psychologist to make a first independent assessment some time before the specific request was made. The independent physician gave the patient a psychological examination to ascertain whether her request was due to depression. He found he could well understand the patient’s wish for euthanasia and felt it was in keeping with her view of life and her background. It was not due to depression.*

*Then, two months before the patient requested that her life be terminated, the physician had asked a SCEN physician to make a second independent assessment. During an interview with this second independent physician, the patient said that, given the protracted nature of the disease, she was afraid she no longer had any real future and would become isolated. She said she preferred to stay in bed in the mornings because she no longer felt able to do anything. She described the despair she felt as she carried out her activities of daily living. It took her a very long time to get anything done. She had had to give up all her hobbies and stop reading and watching television, as she could no longer concentrate. She also said she regularly felt physical discomfort, although she was unable to describe the feeling in detail. The despair she felt in her daily life made the world seem menacing to her. Her suffering had become virtually unbearable to her, which the independent physician found understandable. He concluded that her request had been voluntary and well-considered. The patient said she was not yet ready to have the procedure performed.*

*The second independent physician saw the patient a second time, two weeks before her life was terminated and after she had specifically requested euthanasia.*

*This time the attending physician had asked the independent physician to focus on whether the patient was capable of making an informed decision. The independent physician was satisfied that she was, and concluded that all the due care criteria had been fulfilled.*

*Finally, the attending physician consulted a third independent physician, a clinical geriatrician, and again asked him to assess the patient’s decisional competence. This independent physician saw the patient two weeks before her life was terminated.*

*The third independent physician’s report stated that the patient had sometimes had trouble expressing herself, but that she had made reasonably clear what she wanted to say. She repeatedly indicated that she could no longer cope with her current situation and with having to live with Alzheimer’s disease. She was expected to live about six more years, and saw this as a heavy and intolerable burden. The independent physician found that, despite having Alzheimer’s disease, she could still provide a reasonable justification for her request for euthanasia. She could grasp the implications of her decision, and did not feel that any of the alternatives would improve the quality of her life. The independent physician could see no reason not to grant her request. The patient had been decisionally competent when she made the request.*

*The committee found that the physician had acted in accordance with the due care criteria.*

**Case 2009-4: Dementia**

**The physician consulted several experts in order to establish whether the patient was decisionally competent, and the request for termination of life voluntary and well-considered.**

*The patient, a man in his 70s, had been diagnosed with Alzheimer’s disease. Repeated neurological and neuropsychological examinations had revealed that, since 2004, he had been suffering from a slow and progressive dementia syndrome with clinical symptoms of Alzheimer’s. The patient was suffering unbearably due to the fact that he had begun to lose his grasp of matters and his grip on life, and to the realisation that he was in a process of decline. He found the lack of prospects for the future and the loss of dignity and control unbearable. Four and a half months prior to his death the patient had made his first specific request for euthanasia to his physician. Since that time, he had repeated the request on many occasions. He had previously discussed the possibility of euthanasia when it had become clear he was suffering from dementia, and had often discussed his desire for euthanasia with his wife and children. He had been thinking seriously about euthanasia for about a year, and had made an advance directive. Prior to the legally required independent assessment, the physician had approached the neurologist treating the patient and a psychiatrist as experts. An independent general practitioner, who is also a SCEN physician, was consulted as an independent physician. The psychiatrist first visited the patient five months before his death, and had had contact with him several times after that.*

*The neurologist wrote in his report that the patient was suffering unbearably from his mental deterioration, with no prospect of improvement. He was battling against the loss of his grasp of matters, but it was clear to him that his efforts were to no avail. The patient wanted to retain his dignity. He no longer had the will to live. He was able to do less and less and ‘knew less and less’. As a younger man, the patient had already stated that he would not wish to experience any process of mental deterioration. According to his doctor, this was typical of his down-to-earth, rational character. Previously in the course of his illness, the patient had indicated that he would want to have euthanasia if his suffering became unbearable. He had made an advance directive in 2008. Eventually, he made a specific request for euthanasia. In his visits to the patient, it had become clear to the psychiatrist that the patient was aware of his cognitive decline. The patient was afraid of what the future would bring, and did not want to suffer a complete loss of dignity. He had always had great aspirations, both in his work and in his associations with others. He was suffering greatly from the fact that there was something wrong with him, that he kept failing, and that he no longer had control. He was afraid that if he could no longer express his desire for euthanasia he would not receive any help to end his life. He was gradually losing his capacities. Sometimes he lost his way. He was afraid of the progressive nature of the illness and of not knowing how he would end up. He was in low spirits, but indicated that he understood that this was a symptom of Alzheimer’s. He was withdrawing more and more from social activities and beginning to lose interest in what was going on around him. He had lost his energy.*

*The patient felt his life was empty and could find no inner peace. He preferred to stay at home. He felt his situation was hopeless and that things were getting worse. He was aware that his memory was failing and that he was being treated more and more as a person with a cognitive disorder, something which he found very uncomfortable. He had no future. He did not want to lose his dignity, nor did he wish to experience an irreversible loss of control over his life. He wanted to die before he was no longer able to recognise his family, and did not wish to become entirely dependent on the help of others. His wish to die and to escape the humiliation of dementia was greater than his desire to see his grandchildren grow up. The patient wanted to die as soon as possible, and his desire for euthanasia remained equally strong every time the psychiatrist discussed the matter with him.*

*The psychiatrist concluded that the patient was decisionally competent and had an abiding wish to end his life in a dignified way before dementia made it impossible for him to recognize his family, who would have to watch him suffer a humiliating decline. The psychiatrist*

*did not believe that the patient would change his mind on the matter. According to the psychiatrist, he had an above-average awareness and understanding of his progressive cognitive*

*disorder and decline in function. Because of his personality, he suffered more than the average in the face of these facts. The psychiatrist noted that the patient’s mood disorder stemmed from the fact that he had a progressively more demeaning condition which he could not escape. The patient was increasingly afraid that there would come a point where he could no longer express his desire for euthanasia, and that it would not therefore be granted. He had already made it clear that he would not wish to experience the entire process of dementia before he became ill, and had remained steadfast in this opinion. The patient had made his request for euthanasia independently, under no pressure from others. The independent physician was of the opinion that the notifying physician had conducted the euthanasia process with due care. The physician had known the patient for a long time.*

*He had consulted a psychiatrist to assess his decisional competence, and asked the psychiatrist and the neurologist to explore whether any other treatment options were available,*

*including counselling, antidepressants and the like. A number of antidepressants had been tried, but none had had any clear effect. The independent physician concluded that the patient had made a voluntary and well-considered request for euthanasia. The psychiatrist had found him to be decisionally competent. The independent physician was of the opinion that the due care criteria had been complied with. The committee found in its assessment of this case that, in patients suffering from dementia, greater caution must be exercised in responding to any request for termination of life. The nature of their condition can after all give rise to doubts as to whether dementia patients are decisionally competent, and whether their request is voluntary and well-considered. The question of whether their suffering is unbearable is also relevant. The committee was of the opinion that the physician had at any rate proceeded with great caution, not only consulting an independent physician, but also seeking the expert opinion of the neurologist treating his patient and a psychiatrist, who had based their opinion of his request for euthanasia and the nature of his suffering on their own expertise. The committee found that the physician had acted in accordance with the due care criteria.*

**Case 2010-7**

**The physician could make it clear that he found an Alzheimer patient’s suffering palpably unbearable.**

**Finding: criteria complied with**

*Since mid-2009 the patient, a woman in her eighties, had felt that she was becoming forgetful. She had to write things down in order to remember them, but soon after reading them she had forgotten them again. Watching television was also more and more difficult, as it was all getting too fast for her. In early 2010, at her own request, she was examined by a psychiatrist, who found her to be decisionally competent. She then underwent a neuropsychological examination to determine whether there was cognitive deterioration. Several functional disorders and general cognitive deterioration were found, and it was concluded that she was suffering from incipient dementia, possibly Alzheimer’s disease. This was confirmed by a geriatrician who was consulted by the attending physician. The patient suffered from the future prospect of humiliation and loss of dignity. She was already losing her sense of time, and saying the same things twice. She realised she was already suffering from slight dementia, and was afraid of a further decline which, among other things, would lead her to become dependent and incontinent, and to lose her way. She was also afraid of being unable to request euthanasia because she was no longer decisionally competent. She wanted to die with dignity, while she ‘still had all her faculties’.*

*The patient had experienced at close hand what Alzheimer’s disease could be like. Her mother, sisters and brother had all had the disease in later life, and had eventually died in nursing homes. Whenever she had gone to visit them there, she had felt sad and helpless. The loss of dignity that accompanied the disease had made a deep impression on her. She had perceived the way in which people were looked after in nursing homes as degrading. By exercising her brain the patient had done what she could to prevent dementia. She did not want to experience undignified deterioration as a result of dementia, and had therefore signed an advance directive back in 1993. Her disease was incurable, and there was no prospect of improvement in her suffering. This was unbearable to her.*

*She had always told the physician that she wanted euthanasia if she developed dementia. Some six weeks before she died, when it was becoming increasingly clear that she was suffering from dementia, she asked the physician for euthanasia, and she repeated her request several times thereafter. In addition to the earlier advance directive, she had also signed a recent one. At the physician’s request, one of her daughters had also written down the story she had told of her own life.*

*According to the physician there was no pressure on her from those around her, and she was aware of the implications of her request and her physical situation. This had been confirmed by a psychiatrist.*

*An independent specialist, who was also a SCEN physician, was consulted as an independent physician. He saw the patient just over a week before she died. According to his report she was lucid during the interview. She told him of her long-time fear of developing dementia and her experience with her relatives who had suffered from it. She felt disillusioned: she had developed the disease despite her efforts to keep abreast of things and exercise her brain. She was afraid of the future, since she knew exactly what was going to happen to her. Physically, too, she had deteriorated a great deal. Since she was unsteady on her feet, she could no longer walk her dog, and she was finding stairs more and more difficult. She was afraid of falling while going to the lavatory at night and not being able to alert anyone because she had forgotten to put her alarm on. She was still living alone, but her daughters took turns to stay with her because of her fear and panic at losing her sense of time. She was afraid of losing touch with reality before long and eventually being unable to request euthanasia because she was no longer decisionally competent. She did not want to experience the total humiliation and loss of dignity that the disease would eventually lead to. The independent physician’s report confirmed that the patient’s suffering was unbearable with no prospect of improvement, and stated that her fear of the future was realistic. There were no alternative ways to alleviate her suffering.*

*The committee found that the physician could be satisfied that the patient’s request was voluntary and well-considered, that her suffering was unbearable with no prospect of improvement, and that the physician had acted in accordance with the statutory due care criteria.*

**Case 2011-6**

**Voluntary and well-considered request from a patient with Lewy body dementia**

**Finding: criteria complied with**

*In the years before his death, a man in his fifties developed progressive memory problems. Two years before his death, he was diagnosed with Lewy body dementia, a condition for which there is no cure. Six months after this diagnosis, it was confirmed by a second opinion requested by the attending physician. Despite attempts to slow down the disease, the patient’s cognitive functions deteriorated progressively. He began to develop choreic movements and experience hallucinations, which made him sleep poorly. The patient’s suffering was caused by consciously experiencing his own decline, the progressive deterioration of his cognitive functions, very realistic visual hallucinations and continual, severe, choreic movements. He also suffered from the knowledge that his situation would only worsen and that he might have to be admitted to a nursing home in the future. It was palpable to the attending physician that the patient’s suffering was unbearable to him. His suffering was clearly without prospect of improvement.*

*The documents make it clear that the attending physician and specialists gave the patient sufficient information about his situation and prognosis.*

*Two months before his death the patient discussed the circumstances in which he would want euthanasia with his attending physician. They spoke about euthanasia again a number of times after that occasion. A few days before he died, the patient specifically requested euthanasia and repeated this request several times. There were several advance directives. An independent physician (a specialist who was also a SCEN physician) saw the patient two weeks before his death. According to her report, the patient gave an impression of old age, responded slowly, spoke haltingly and initially made only brief eye contact. He was visibly restless and tense. At his wife’s suggestion, he sat down on the sofa. During their conversation, the patient clearly described the uncertainty and sadness that developing this condition at his age had caused him. He knew the dementia now largely determined his life. He knew that there was no treatment that would allow him to live a little longer with at least some dignity. He wanted to stay in charge of his situation and had dictated his wishes to his wife, who had written them down for him. He was aware of his changing cognitive capacity and of the other limitations that, among other things, prevented him from working, driving and riding a motorcycle. These cognitive and physical limitations would only worsen. He absolutely did not want to be admitted to a nursing home or any other institution and he knew the time was coming that this would become unavoidable. He did not want to become incapable of recognising his wife and children. He was exhibiting increasing impulsivity as a result of his demential syndrome, requiring him to be restrained by his wife. He knew that these behaviours would only get worse, and experienced this as degrading.*

*According to the independent physician, the patient had a clear opinion about when he would want euthanasia to be performed and he had discussed this at length with his physician and close family. The patient was decisionally competent during this conversation. His request was voluntary and well-considered. The independent physician believed that the patient’s condition was untreatable and he was already at a stage that it was almost impossible for him to live with dignity. The patient’s suffering was palpably unbearable. It was caused not only by his physical decline, fears and hallucinations but also by the knowledge that further deterioration, physically, cognitively and behaviourally, was inevitable.*

*The independent physician was convinced that the due care criteria would be fulfilled at the point where the patient actually requested the euthanasia procedure to be performed, provided this took place within six weeks. But if euthanasia were not performed within six weeks, the attending physician would have to consult an independent physician again. In reviewing this notification, the committee considered that a request for termination of life from a patient suffering from progressive dementia must be responded to with even greater care than usual. There may be doubts about whether the patient is decisionally competent, and whether the request is voluntary and well-considered. It is also necessary to ascertain whether the patient’s suffering is in fact unbearable. In the committee’s opinion, the attending physician acted with due care in this case.*

*A detailed advance directive, dictated by the patient and describing his suffering and his wishes, was included in the records. The patient’s records show that the patient’s wish for euthanasia in the event of unbearable suffering had existed for some time, and that he had arrived at a point where he wanted his wish to be carried out because his suffering had become unbearable to him. The records also revealed that the patient remained oriented to time, place and self. The independent physician, after discussing the patient with the attending physician, examining relevant documents and extensively interviewing the patient concluded that she had no doubts about the unbearableness of the patient’s suffering and his decisional competence. The independent physician was a geriatrician which the committee considered made her opinion sufficiently authorititative.*

*In view of the above facts and circumstances, the committee found that the due care criteria had been complied with.*

**Case 2012-4**

**Finding: criteria complied with**

**Summary: patient with advanced dementia. The physician established satisfactorily that**

**the patient’s suffering was unbearable to her and without prospect of improvement. There**

**was no reasonable alternative in the patient’s situation. The physician was satisfied that her**

**request was voluntary and well-considered.**

*The patient, a woman in her eighties, was first diagnosed with dementia in spring 2010. The most prominent symptoms in her case were paranoid delusions and hallucinations. The patient had a long history of severe osteoporosis, which worsened in recent years, leading to vertebral collapse and multiple fractures and causing the patient severe pain. Just over a month before her death, the patient had a fall, breaking her hip and wrist, and required surgery. Her condition was incurable. She could only be treated palliatively. Despite the use of various types of analgesics the patient was not free from pain. Nor were the two types of antipsychotic drug that she was administered effective. In the last few weeks before her death, after she had been discharged from hospital, the patient suffered from severe paranoid delusions, anxiety and confusion. This also made her physically restless, so that she fell several times in the last week, even though she was receiving round-the-clock care. There was no prospect of improvement in her situation.*

*The patient, who had always set great store by her independence and self-reliance, was suffering primarily from the pain, which was difficult to manage, but also from her lack of mobility as a result of her deteriorating condition. She also suffered from the knowledge that she had progressive dementia. She was familiar with the process of cognitive decline, as she had cared full-time for her husband, who suffered from dementia, for a number of years. She was afraid that, like her husband at the time, she would eventually need to be admitted to a nursing*

*home, an event she did not want to go through herself.*

*The patient also suffered enormously from the paranoid delusions which, especially in the final weeks of her life, made her very fearful. The patient found her suffering unbearable.*

*Apart from the palliative measures that had already been taken, there were no other means acceptable to the patient to alleviate her suffering. She did not want to be admitted to a nursing home which, in any case, would be unable to prevent her from falling. She was too restless and suspicious to be fitted with a morphine pump.*

*The patient’s request*

*When the patient first registered with the physician’s practice in 2004, they talked about euthanasia in general terms. At the beginning of 2011 she gave the physician an advance directive, which stated specifically that she wanted her life to be terminated should she ever be facing the same situation as her husband was in at that time.*

*About three months before her death, the patient told her physician that she wanted her life to be terminated when her suffering became unbearable to her, a stage she did not consider*

*herself to have reached at that time. She again referred to her husband’s situation and emphasized that she did not want anything like that to happen to her.*

*About two weeks before her death, the patient’s children specifically requested that her life be terminated. The patient herself had indicated indirectly that she wanted to die, saying things like ‘I don’t want to live this way any longer’ and ‘I can’t take it any more’. At some point she refused to take her medication because she ‘wanted to die anyway’.*

*According to the physician, the voluntariness of the patient’s request was evident from, in particular, the many occasions that the patient had discussed her wish for euthanasia with her. The physician did not believe the patient was influenced by or under pressure from others to make her request.*

*The physician also found the request to be well-considered because the patient had discussed her wish for euthanasia a number of times when she had still been lucid, and had*

*been well aware at the time of the implications of her request and her physical condition.*

*In the final weeks before her death, an in-depth talk with the patient was no longer possible. However the day before the patient’s death, the physician had used the word ‘euthanasia’ in a conversation with the patient and she had had the impression that the patient understood what she was talking about. The patient had then indicated she had lived long enough and had had a good life. The physician’s decision was also based on the patient’s behaviour and*

*things she had said in the weeks before her death. This included thanking the physician profusely for everything she had done for her and saying goodbye to her loved ones. The physician also relied on the opinions of the SCEN physician and of the nurse who had cared for the patient in the last two days of the patient’s life. The SCEN physician’s impression from conversations with the patient was that the latter had a wish to die; the nurse believed the patient was suffering greatly.*

*The SCEN physician saw the patient twice. In his report he concluded unconditionally that all the due care criteria had been complied with.*

*Voluntary, well-considered request*

*In reviewing this notification, the committee observed that a request for termination of life from a patient suffering from progressive dementia must be responded to with even greater care than usual. In view of the nature of the condition, there may be doubts about whether the patient is decisionally competent, and whether the request is voluntary and well-considered.*

*Under section 2 (2) of the Act, a physician can carry out a patient’s request for euthanasia in cases where the patient is no longer capable of expressing his wishes, provided the patient laid down these wishes in an advance directive when he was still competent to make a reasonable appraisal of his own interests. The due care criteria likewise apply here.*

*In this case, it could be established that the patient had discussed her wish for euthanasia several times in the years that her cognitive functioning was still unimpaired. She had also given her physician an advance directive in 2011, explaining that she wanted her life to be terminated when admission to a nursing home became unavoidable. Three months before her death, the patient had given the physician to understand that she wanted her life to be terminated if her suffering became unbearable. She also referred to the situation in which her husband eventually found himself: his psychogeriatric symptoms resulted in him being admitted to a nursing home. This was a situation she emphatically did not want to experience. In subsequent months, the patient and her physician no longer discussed euthanasia.*

*In the weeks before her death – after she had been discharged from hospital – the patient*

*was no longer able to put her wish into words as such, but she did make it clear that she wanted to die. According to the doctor she had said she ‘didn’t want to live this way any longer’ and ‘couldn’t take it any more’. She also refused to take her medication because she ‘wanted to die anyway’. In the weeks before her death she had thanked the physician profusely and said goodbye to her loved ones. On the evening the procedure was carried out the patient had been unusually calm. When the physician said she was going to give her a small injection, the patient had expressed her acquiescence.*

*On the question of whether the patient’s request was voluntary and well-considered, the committee noted that, although the patient could not request euthanasia in so many words, her behaviour and things she had said until just before her death made it clear that she wanted to die because of her pain, her forgetfulness and because she did not want to be put in a nursing home. The physician established satisfactorily that she had become convinced that the patient’s wish to die was in complete accordance with the patient’s wish for euthanasia, as previously expressed both orally and in writing.*

*In view of the above facts and circumstances, the committee found that the attending physician could be satisfied that the patient was decisionally competent when she drew up her advance directive and that her request for euthanasia was voluntary and well-considered.*

*Unbearable suffering with no prospect of improvement*

*On the issues of unbearable suffering with no prospect of improvement, information provided*

*to the patient and acceptable alternatives, the committee held as follows.*

*On the advice of an external expert, the physician administered Seroquel for a number of days in an attempt to relieve the patient’s complaints, but she thought she should not wait any longer for beneficial effects to arise because of the sharp deterioration in the patient’s condition. The patient was not only in great pain, she was also very anxious and had frequent panic attacks. She was very restless and at increased risk of falling. She had also lost control over her bowel movements.*

*The physician established satisfactorily that the patient’s suffering was unbearable to her.*

*The independent physician consulted had also concluded that the patient’s suffering was palpably unbearable. Admission to a nursing home was not a reasonable alternative as, there too, the patient would be at increased risk of falling. Moreover, when the patient had still been able to communicate clearly, she had said several times that a nursing home represented unbearable suffering for her.*

*In view of these facts and circumstances, the committee found that the attending physician*

*could be satisfied that the patient’s suffering was unbearable and without prospect of improvement. The physician gave the patient sufficient information about her situation and prognosis. Together, the physician and the patient could be satisfied that there was no reasonable alternative in the patient’s situation.*

**Case 2013-6**

**Finding: criteria complied with**

**Summary: decisional competence of an Alzheimer’s patient.**

*The patient, a woman in her eighties, had been diagnosed with Alzheimer’s disease three years before her death. She was being monitored by a neurologist and did not want to be assisted by a psychogeriatric team. Until recently, the patient had been able to enjoy everyday activities, such as cycling, walking and doing puzzles. These activities had compensated for the loss of depth in personal conversations. She was now deteriorating, however. The patient suffered from restlessness, including at night, insomnia, memory loss, aggressive mood swings, sadness and her complete dependence on care. She felt unwell and lightheaded. Sometimes she would not recognise her family. She was desperate and was suffering from a realistic fear of the suffering that lay ahead of her. Six years before her death, the patient had already discussed euthanasia in general terms with her physician. Shortly after her diagnosis she discussed her euthanasia wish again. She had witnessed the sickbed of a family member with Alzheimer’s and absolutely did not want to go through that herself. She flatly refused to go into a nursing home. The patient discussed her euthanasia wish several times with her attending specialist. She also had an advance directive, dated four years earlier, and a document in which she gave a family member power of attorney to make decisions for her if she was unable to do so herself. In these documents she explicitly mentioned her euthanasia request. Lastly, there was a video recording dating back to two years before her death, in which the patient confirmed her euthanasia wish orally. In all these expressions of her wishes, she named going into a nursing home as the ultimate boundary: if that were to become necessary, she wanted euthanasia.*

*Three weeks before her death, the patient asked her GP to perform the euthanasia procedure. Every time she requested euthanasia, she would remove her wedding ring and her watch. She did this on every occasion until she died. The physician was convinced that her request was voluntary and well-considered. The GP consulted a SCEN physician as the independent physician. The SCEN physician took note of all the information about the patient and her euthanasia request, including the video recording. During the interview with the patient, the independent physician noted that she was consistent in her wish for euthanasia, but that she had difficulty answering other questions. Initially, the independent physician concluded that the patient was decisionally incompetent at the time of the interview. He qualified that conclusion when giving a personal explanation to the committee. According to the independent physician, the patient was decisionally competent with regard to her request for termination of life, but incompetent in all other areas. He also considered the patient to have been decisionally competent when she wrote the advance directive and recorded the video. The independent physician found that the due care criteria had been complied with.*

*Two of the patient’s family members were present when euthanasia was performed. Despite her increasing dysphasia, the patient was able until the end to communicate the essence of her wish to be allowed to die to her family as well. Before performing euthanasia, the physician asked the patient again what she wanted. She confirmed that she wanted euthanasia and was aware that the euthanatics would be administered.*

*The attending physician was later invited to an interview with the committee, where he explained that it was possible, if you did not know the patient well, to doubt her decisional competence, because the patient needed rest and concentration to be able to communicate verbally. The physician was afraid that if her illness progressed further she would no longer be able to repeat her request. In response to questions from the committee, the independent physician said that he had doubts in particular about the patient’s decisional competence in other areas. He had no doubts as to her decisional competence with regard to her euthanasia request. He based this in part on the patient’s decisional competence when she drew up the advance directive, recorded the video and ‘maintained’ the written request as was apparent from the file.*

*The patient knew why the independent physician was there and was able to make clear how much she was suffering from losing control of the situation, and that she did not want to live like that any longer. The committee found, partly on the basis of the explanations given by the attending physician and the independent physician, that the statutory due care criteria had been satisfied.*

**Case 2014-35**

**FINDING: due care criteria complied with**

**DUE CARE CRITERIA: voluntary and well-considered request, consulting an independent physician**

**KEY POINTS: dementia, role of the advance directive**

**SUMMARY: The patient, a woman in her seventies, suffered from Alzheimer’s disease. The physician and the patient had repeatedly discussed euthanasia ever since she was diagnosed. The patient had drawn up a detailed and updated advance directive, with a provision on dementia. Right until the end she was able to express her euthanasia request to the physician, though not necessarily in words. The patient’s suffering as observed by the physician matched what she had previously described orally and in the advance directive as unbearable to her. The patient could not express her request orally to the independent physician, but the independent physician was able to rely on the advance directive. The physician could be satisfied that the patient’s request was voluntary and well-considered, and that her suffering was unbearable without prospect of improvement.**

*More than a year before her death, the patient, a woman in her seventies, was diagnosed with Alzheimer’s disease. Her condition was incurable. She could only be treated palliatively. She was given medication. The patient and her family were supported by a dementia case manager (dementia case managers give professional advice and information and provide support to dementia patients and their families). The patient’s cognitive and motor skills deteriorated rapidly. Two months before her death, the patient began receiving personal care at home. At that time the patient also started to go to day care.*

*The patient’s suffering consisted of the fact that she was no longer able to function independently and needed help with everything. She had always been an independent person and the loss of control over her life made her very sad. When day care and home care became necessary, the patient had had enough. She had seen people around her become incapacitated as a result of Alzheimer’s disease and she did not want to go through that process herself. The physician had known the patient for years and, partly in view of the patient’s personality before her illness, was satisfied that the patient’s suffering was unbearable to her. The patient’s suffering was without prospect of improvement according to prevailing medical opinion.*

*The patient had discussed euthanasia with the physician before. Around 10 months before the termination of life was performed, the patient handed the physician an advance directive, including a provision on dementia.*

*A month before her death, the patient asked the physician to actually perform euthanasia. When speaking to the physician, the patient proved to have an understanding of her illness. She substantiated her request for euthanasia with reasons. The patient subsequently repeated her request to the physician on several occasions.*

*The physician was satisfied that the patient understood what euthanasia entailed, right up to the end, and that termination of her life was her express wish.*

*The physician consulted two independent physicians who were general practitioners and independent SCEN physicians. The first independent physician saw the patient two months before the termination of life was performed, after he had been informed of the patient’s situation by the physician and had examined her medical records. At that time the patient had not yet requested that euthanasia be performed. In the opinion of the first independent physician, assessment of compliance with the due care criteria was not yet necessary.*

*The second independent physician saw the patient a month before the termination of life was performed, after she had been informed of the patient’s situation by the physician and had examined her medical records. In her report the independent physician gave a summary of the patient’s medical history and the nature of her suffering. The second independent physician concluded that the patient understood what euthanasia entailed and that she had requested euthanasia because she had dementia. The independent physician had doubts, however, as to the patient’s decisional competence in relation to her current wish to die. The patient was unable to consistently express at what moment in time she wanted euthanasia to be performed. In addition, her understanding of her illness varied depending on the degree*

*of fatigue or agitation and the phase of the disease. The independent physician advised the physician to consult a psychogeriatric physician to have the patient’s decisional competence assessed.*

*Three days after the second independent physician’s visit, a psychogeriatric physician visited the patient. According to the psychogeriatric physician the patient understood what euthanasia was and could say that a situation could arise in which she would request euthanasia. The patient was unable to specify what that situation would be. When her children talked about accepting home care and day care, however, she rejected this idea with vehemence and anger. The patient did not make a concrete request for euthanasia and could not remember having done so previously. She did say, however, that there were times when she thought life was no longer worth living and she would rather be dead. She could imagine requesting euthanasia one day.*

*Partly on the basis of the findings of the psychogeriatric physician, the second independent physician concluded that due to her loss of any concept of time the patient was no longer able to formulate a specific moment when the termination of life procedure was to be carried out. According to the second independent physician, the well-documented advance directive could replace the patient’s oral consent. The independent physician concluded, partly on the basis of her interview with the patient, that the due care criteria had been met.*

*From the notification, it was insufficiently clear to the committee how the physician had ascertained that the patient’s suffering was unbearable to her at the time of the termination of life. The committee also had questions about the voluntary and well-considered nature of the patient’s request, partly in view of what the psychogeriatric physician and the SCEN physicians said in their reports.*

*The physician gave an oral explanation, which included the following. The patient was an upper middle class lady who knew exactly what she did and did not want. After she was diagnosed it was already clear to the physician that she would at some point request euthanasia. The physician said that the complicating factor in this patient’s case was the fact that at the end she increasingly lost her sense of time. Her wish for euthanasia was clear, but it was not always clear at what moment she wanted her life to be terminated. On a good day she would know when her birthday was and say that she wanted to die on her birthday. On a bad day she would be confused and unable to express this. Moreover, the patient tended to put on a brave face in front of strangers. In the end, the physician found the case to be very clear and was satisfied that the due care criteria had been complied with.*

*A number of issues were still insufficiently clear to the committee after reading the file and hearing the physician’s oral explanation. For instance, the committee wanted to ask the second independent physician some questions about her findings based on her second visit to the patient, at which the physician was also present. In particular, the committee wanted to hear from the second independent physician what her opinion was, after the second visit, regarding the patient’s decisional competence, the voluntary and well-considered nature of the request and the unbearable nature of her suffering.*

*The independent physician gave an oral explanation, which included the following. The committee had noticed in the patient’s medical record that the independent physician had visited the patient together with another SCEN physician. This was not apparent from the independent physician’s report. When asked about the matter, the independent physician explained that the SCEN physician who had accompanied her worked at the same practice as she did. Although the independent physician had been a SCEN physician for a long time, she had never before had to assess compliance with the due care criteria for a patient with dementia. She wanted to exercise the greatest possible care and she felt supported by the presence of her colleague, who had specific expertise on euthanasia and dementia. The colleague did not take part in the conversation with the patient, but the independent physician and her colleague did discuss the case afterwards. The independent physician had not reported this course of events because she did not think it was relevant to the report. In the end the independent physician based her findings on the conversation with the patient, the conversation with the physician, the medical file, the assessment by the psychogeriatric physician and the patient’s advance directive. The provision on dementia (drawn up 10*

*months before the patient’s death) clearly stated what the patient absolutely did not want and what would be unbearable suffering for her. The independent physician was told that at that point the patient was already losing her sense of time. The independent physician could see that the patient was suffering, while the unbearable nature of the suffering was clear to her in part from the way the whole process had gone. The patient’s situation matched the situation she had described in her advance directive as never wanting to experience. Her dependence and the loss of autonomy formed the key components of the unbearable suffering. What contributed to the unbearable nature of the suffering was the fear of future suffering; her*

*symptoms were only going to get worse.*

*The committee initially questioned how the physician had become convinced that the patient’s request for euthanasia was voluntary and well-considered and that she was suffering unbearably. It was clear from the file that at the end the patient was no longer able to properly express and substantiate her request orally, partly due to her lack of a sense of time caused by her illness. The oral explanations by the physician and the second independent physician made the course of events clear to the committee. The voluntary and well-considered nature of the request was mainly clear from the fact that the physician and the patient had discussed it from the moment she was diagnosed. The physician documented this process carefully in the patient’s medical record. Moreover, at an earlier stage the patient had signed and handed*

*over a detailed advance directive to the physician. The physician also said that the patient was able to indicate her request to the physician right to the last moment, though not necessarily in words. According to the physician this is not uncommon in patients with dementia. Under stress and social pressure, and as a result of fluctuations in the severity of symptoms, their decisional competence and/or communication skills may vary. On several occasions the physician saw the patient become angry and distressed when she realised that she had lost her autonomy and become dependent. That suffering matched the suffering that the patient had previously expressly indicated, both orally and in the advance directive, as being unbearable to her.*

*The independent physician based her findings on her own observations during her interview with the patient, but also on the entire process from the time of diagnosis, her knowledge of which was based on the medical records and conversations with the people involved. The independent physician felt she was supported by the psychogeriatric physician’s findings. Although the patient could not express her request orally to the independent physician, the independent physician was able to rely on the patient’s advance directive in this case. The advance directive included the patient’s name, was dated and signed and had been updated and discussed with the physician regularly. It stated clearly what the patient would consider to be unbearable suffering.*

**Case 2015-107**

**FINDING: DUE CARE CRITERIA COMPLIED WITH**

**DUE CARE CRITERIA: voluntary and well-considered request, no prospect of improvement, unbearable suffering, no reasonable alternative**

**KEY POINTS: advanced dementia, role of the advance directive**

**SUMMARY: A woman with Alzheimer’s disease was suffering unbearably from cognitive deterioration, impaired practical, executive and phatic functioning, and growing dependence. When admission to a nursing home seemed inevitable, she wanted euthanasia. There was an updated advance directive. The physician affiliated with the End-of-Life Clinic (SLK) followed the patient for a long period and consulted a geriatrician and two independent physicians. It was clear to the second independent physician, who was an elderly-care specialist, that the patient’s wish for euthanasia was current. The physician could be satisfied that the patient’s request was voluntary and well-considered, that her suffering was unbearable, and that there was no reasonable alternative.**

*Around three years before her death, the patient, a woman in her seventies, was diagnosed with dementia (Alzheimer’s disease). The patient refused day care, a case manager and check-ups by the geriatrician.*

*The patient’s suffering consisted of cognitive deterioration, impaired phatic, practical and executive functioning, loss of control over her thoughts and actions, and growing dependence on her husband’s care. One of her parents had had dementia and had gone into a nursing home, where they had mostly sat crying. The patient had always said that she thought this was degrading and humiliating, that she would never want to be in that situation of dependence and sadness herself, and that she never wanted to go into a nursing home. Because her husband could no longer care for her properly, she had been assessed as requiring nursing home care and admission was imminent. She was suffering unbearably from the absence of any prospect of improvement in her situation, from fear and uncertainty, and aversion to experiencing a process of deterioration like her parent’s and to being taken into a nursing home.*

*The patient had discussed euthanasia with her general practitioner before. As her general practitioner could not comply with her euthanasia request, she contacted the End-of-Life Clinic (SLK). The physician affiliated with the SLK was in contact with the patient regarding her wish for euthanasia for more than two and a half years. She visited the patient several times and maintained email contact with the patient’s husband.*

*The physician consulted a geriatrician, who examined the patient more than a month before her death to assess her decisional competence in relation to her wish for euthanasia. The geriatrician established that the patient was in an advanced stage of dementia and that there were no signs of an underlying mood disorder. However, she was unable to form a judgment on the patient’s decisional competence in relation to her wish for euthanasia, because at no point did the patient spontaneously express a desire to die.*

*Three and a half weeks before her death, the patient asked the physician to perform the procedure to terminate her life soon. Her husband could no longer care for her properly and as a result the patient would have to go into a nursing home. She indicated that she did not want this; in such circumstances she wanted to die. The physician found that the patient was decisionally competent and the request was voluntary and well-considered.*

*The physician consulted two independent physicians who were also SCEN physicians. The first visited the patient three weeks before the termination of life was performed.*

*According to the first independent physician, the patient showed symptoms of an advanced stage of dementia. Her request for euthanasia in the event that she would have to go into a nursing home proved consistent and she was able to substantiate it. The patient was equally consistent in her opinion that she did not want to request euthanasia at that point in time. The independent physician concluded, partly on the basis of his interview with the patient, that the due care criteria had not been met because she did not have a current wish for euthanasia. He advised the physician to consult a SCEN physician with more specific expertise and contacted a colleague in his peer supervision group, an elderly-care specialist, who was willing to assess the patient soon after. The physician followed his advice.*

*The second independent physician, an elderly-care specialist, visited the patient 11 days before the procedure to terminate her life was performed, after she had been told about the patient’s situation by the physician and had examined her medical records. According to this independent physician, the patient indicated that she kept losing things and that she needed more and more help; this caused her great distress. When the independent physician and the patient subsequently talked about the future, the independent physician said that with dementia – the patient was familiar with the word – the expectation was that it would only get worse: it was a brain disease with no prospect of improvement. At that moment, the patient spontaneously said ‘But I’ve had enough’ and ‘I don’t want to go on’. When asked by the independent physician what she’d had enough of, she said ‘Everything, all the things I can’t do anymore, more and more things’. When asked what she meant when she said she did not want to go on, she said ‘I want to die’. When the independent physician repeated the question, asking if she wanted to die now, she replied ‘Yes, I want to die’. She said these things forcefully, with conviction and spontaneously, without any contribution from her husband. It was clear that her suffering was severe and that ‘wanting to die’ was now a current reality for her.*

*The independent physician was satisfied that the request came from the patient herself, also in view of the fact that she expressed her desire to die with conviction and on her own initiative during the interview. The independent physician found the request to be wellconsidered, in view of the previously documented interviews and the long-term guidance she had sought and received from the physician.*

*The second independent physician concluded, partly on the basis of her interview with the patient, that the due care criteria had been met.*

*The physician performed the procedure for assisted suicide. The patient took the beaker with the barbiturate potion from the physician and drank it, even though it could be seen that she did not like the taste.*

*The committee noted the following in connection with the request being voluntary and well-considered. The file contained the patient’s advance directive, with a special clause on ‘dementia‘, which had been signed in August 2009 and reaffirmed several times since then.*

*In January 2013, eight months before she was diagnosed with Alzheimer’s disease, the patient drew up an advance directive, in which she stated that the process of dementia in one of her parents had particularly affected her, was never out of her mind and was of great influence on her opinions on growing old and being old. From that time onwards, she had made it clear, orally and in writing, that she did not want to end her life that way. For her, losing her dignity, losing contact with her loved ones, being dependent and being put away would be to suffer unbearably. She wanted to stay in her home as long as possible, where her husband would be her carer. If that were no longer possible, due to deteriorating mental and/or physical circumstances, then for her it would be time for a voluntary, selfdetermined and dignified end to her life.*

*The patient contacted the SLK in January 2013, after which the physician visited her for the first time. The physician agreed with the patient that her husband would keep an eye on when the moment for euthanasia was approaching and that the physician would maintain email contact with him. Over the course of two and a half years, the physician visited the patient on several occasions and maintained contact with her and her husband via email. Just over seven weeks before her death, the patient’s husband indicated that, given her deteriorating condition, caring for her was becoming too difficult for him.*

*At that time, the physician began assessing the euthanasia request and the patient’s current decisional competence in relation to her request. She consulted a geriatrician. The geriatrician could not form a judgment on the patient’s decisional competence in relation to her wish for euthanasia, because at that moment the patient did not express a wish for euthanasia.*

*Three and a half weeks before her death, the patient asked the physician to perform the procedure to terminate her life soon. Her husband could no longer care for her properly and as a result the patient would have to go into a nursing home. She indicated that she did not want this; in such circumstances she wanted to die. The physician found that the patient was decisionally competent and the request was voluntary and well-considered. The physician recorded this conversation on her iPad and made a transcript.*

*The physician then consulted an independent SCEN physician, who found that the patient’s request for euthanasia in the event that she would have to go into a nursing home was consistent and she was able to substantiate it. However, the patient was equally consistent in her opinion that she did not want to request euthanasia at that point in time.*

*He advised the physician to consult a physician with more specific expertise. The physician who was subsequently consulted, an elderly-care specialist and SCEN physician, visited the patient 11 days before her death. The patient indicated spontaneously and clearly to the SCEN physician that it had been enough and she wanted to die. The independent physician was satisfied that the request came from the patient herself, also in view of the fact that she expressed her desire to die with conviction and on her own initiative during the interview. The independent physician found the request to be well-considered, given the previously documented interviews and the long-term guidance she had sought and received from the physician.*

*On the basis of the above, the committee found that the physician could reasonably conclude that the patient’s request was voluntary and well-considered. Although over the course of time there had been moments when the patient did not express a clear desire to die, it emerged that she clearly expressed that desire in the interviews with the physician and the second independent physician – an elderly-care specialist. To them, she indicated unambiguously that she wanted to die now that her husband could not care for her properly anymore and she could no longer live in her own home, in view of her complete dependence on care.*

*The committee notes the following as regards the patient’s suffering being unbearable.*

*In her advance directive, the patient indicated clearly under what circumstances she would experience her suffering as unbearable and would want her life to be terminated. In the many interviews she and her husband had with the physician about her wish for euthanasia, the patient also indicated in detail what unbearable suffering meant for her.*

*When the patient asked for euthanasia to be performed, her suffering was real and current i.e. she was suffering from the loss of control over her thoughts and actions, from fears and uncertainty and complete dependence. At that time the patient was in the situation that she had previously described in her advance directive and in the many interviews with the physician as being one of unbearable suffering.*

*The committee therefore found that the physician could reasonably conclude that at the time when the euthanasia was performed the patient was suffering unbearably.*

*The committee also considered whether there were any reasonable alternatives. After all, home care would have relieved her husband of some of the burden, thus postponing the need for her to go into a nursing home.*

*However, in her advance directive, the patient indicated expressly that her husband would be her carer and that if that were no longer possible, the time would have come for a voluntary and self-determined end to her life. Help from other people was not a reasonable alternative, either for the patient or for her husband. They had always refused any help or care that was offered.*

*Performing euthanasia when her husband could no longer care for her properly was therefore in line with her advance directive. Together, the physician and the patient could be satisfied that there was no reasonable alternative in the patient’s situation.*

**Case 2016-94: early-stage dementia**

**FINDING: due care criteria complied with**

**KEY POINTS: decisional competence, unbearable suffering**

*The patient, a woman in her sixties, became forgetful after suffering a TIA (temporary obstruction of a blood vessel in the brain) in late 2011. In early 2013 she was diagnosed with presenile dementia (dementia at a relatively young age). In the years that followed, the disease progressed and more and more of her daily tasks had to be performed by others. In the autumn of 2015 it became clear that she would soon need daycare. The patient’s suffering consisted of her not being able to function independently anymore and having become fully dependent on others. For instance, she was no longer able to read or write, she had difficulty finishing spoken sentences, she could not drive a car nor could she dress herself. The patient felt trapped in her home. She realised that she was no longer able to take part in society independently and that she had lost control of her life. Having led a very independent life, the patient experienced her suffering as unbearable. The physician was satisfied that this suffering was unbearable to her and with no prospect of improvement according to prevailing medical opinion.*

*From mid-2013 onwards the patient had discussed with the physician the fact that, in due course, she wanted euthanasia. She had also giventhe physician an advance directive. She updated the advance directive several times and added a separate signature to the clause on dementia in the directive. In 2015 she spoke more emphatically about her wish and around two months before her death stated that she wanted euthanasia to be performed within three months. She absolutely did not want to go to daycare outside her home, nor did she want to go into a nursing home. She repeated her specific wish for euthanasia in three subsequent conversations with the physician.*

*The physician concluded that the request was voluntary and wellconsidered. The independent physician stated that the woman was still able to make clear what made her suffering unbearable and that she wanted euthanasia. The committee found that the physician had acted in accordance with the due care criteria.*

**Case 2016-62: advanced dementia**

**FINDING: due care criteria complied with**

**KEY POINT: role of the advance directive in the case of a patient who is decisionally incompetent as a result of advanced dementia**

*In 2005 the patient was diagnosed with dementia (Alzheimer’s disease). He was able to function reasonably well for a number of years, but from 2009 onwards his health deteriorated. In the last year before his death – by now he was in his sixties – his condition deteriorated substantially. The patient had discussed euthanasia with the physician since 2010. In that year he had for the first time written a letter by hand setting out a number of wishes for the future. In 2010 he signed an advance directive, which he supplemented in 2012 after several conversations with the physician, adding a number of more specific circumstances in which he would no longer want to go on living.*

*Those circumstances were described as follows: if he as a person were to change so much that he felt permanently unhappy, if he were to become aggressive and difficult, if he no longer recognised his loved ones, if he were to end up waiting for death, as had a close family member who also had Alzheimer’s disease, if he were unable to take care of himself and became completely dependent on others, if he were suffering unbearably and without prospect of improvement. When he was still able to, the man spoke with both the physician and his family on several occasions about his request for euthanasia at some point in the future, and he updated his advance directive. At a certain point the patient was no longer able to express his request in words, but there were oral and physical expressions that confirmed his wish to die. His wife asked the physician to comply with the written euthanasia request. At that time, his suffering consisted of cognitive problems, apathy, apraxia (difficulty in carrying out actions), agnosia (inability to recognise/name things or persons) and behavioural changes. The patient had become completely dependent on his wife for his personal care. When he woke up in the mornings he was completely disoriented and very sad. He could not remember how to get out of bed. When he was helped with his general daily activities, his facial expressions were of sadness and frustration. He repeatedly indicated he could not and did not want to go on. He was now in a situation in which people expected things of him all day long that he no longer understood. He would then panic, or become startled or angry. He slept a lot. He no longer recognised his children and was no longer aware that he had grandchildren.*

*At the physician’s request an independent elderly-care specialist examined the patient to assess whether he was suffering unbearably. The elderly-care specialist was satisfied that this was indeed the case. According to the specialist, the man was now in the situation that he had previously described as unbearable.*

*The physician was satisfied that this suffering was unbearable to the patient and with no prospect of improvement according to prevailing medical opinion. According to the physician, the situation corresponded entirely with the circumstances the patient had described in his advance directive as not wanting to experience.*

*With regard to the request the committee found that it was clear from the documentation that at the time when euthanasia was performed the patient was no longer decisionally competent. It also found that the physician could be satisfied that the patient was capable of making a reasonable appraisal of his own interests when he drew up his advance directive. In consultation with the physician, the patient regularly updated the advance directive after it had been drawn up and signed. On several occasions he subsequently discussed his wish to die at some point in the future orally with family and physicians. When he was no longer decisionally competent, there were verbal and non-verbal signs that he still wanted his life to be terminated. There were no signs to the contrary. In the committee’s view, the physician had plausibly argued that he was reasonably able to conclude that the patient’s request as worded in the advance directive was voluntary and well-considered.*

*The committee was further of the opinion that, despite the fact the patient was no longer able to describe it himself, the suffering as described in the documentation was evident and fully matched the content of the advance directive. Several factors played a role in the physician’s process of establishing that the man was suffering unbearably: his own observation of the patient, the process of preparing for euthanasia at some point, which took several years and was guided by him with great care, the conversations with the family, the independent physician’s report and the independent elderly-care specialist’s report. The committee found that the physician had exercised particular caution, as is recommended for patients in an advanced stage of dementia. This was clear from, for instance, the fact that in addition to an independent physician he had also consulted an elderly-care specialist, who assessed and described the suffering in a way that enabled the independent physician to conclude that the due care criteria had been complied with. The committee found that the physician had plausibly argued that he was reasonably able to conclude that the patient’s suffering was unbearable and without prospect of improvement. The other due care criteria were also fulfilled.*

**CASE 2016-85**

**FINDING: due care criteria not complied with**

**KEY POINTS: decisionally incompetent patient without a clear advance directive; failure to exercise due medical care. *Note: On September 11, 2019, the physician prosecuted was acquitted.**

*The patient, a woman in her seventies, began to suffer from forgetfulness nine years before her death. Five years later she was diagnosed with dementia (Alzheimer’s disease). A year before her death, the disease began to progress more quickly. She became very anxious, sad and restless. From the afternoon onwards she was sombre, emotional and tearful, and indicated that she wanted to die. When her husband was no longer able to care for her, she was admitted to a nursing home where she had previously gone five times a week for daytime activities.*

*Shortly before she received the dementia diagnosis, the patient had set out her wishes concerning euthanasia in an advance directive and discussed them with her general practitioner and her geriatrician. According to the physicians she was still decisionally competent at thetime. She renewed this advance directive a year before her death. She also discussed this second directive with her general practitioner, who considered her to still be decisionally competent at that time. In both advance directives she indicated that she did not want to be placed in an institution for elderly people with dementia (dementia clause). She stated that she wanted to say goodbye to her loved ones at a sufficiently early stage, in a dignified manner, and that she did not want to experience the process that her mother had gone through in an institution. In the first advance directive she indicated that she wanted euthanasia when she was ‘still to some degree decisionally competent but no longer able to live at home with my husband’. In the second advance directive she wrote that she wanted to make use of the option of euthanasia ‘when I myself think the time is ripe’. The closing sentence read: ‘Trusting that, by the time the quality of my life has become so poor that [...] euthanasia will be performed at my request.’*

*Towards the end of the year before her death, the patient’s condition deteriorated further and at home she often said she wanted to die. Shortly after, she would often say, ‘But not now.’ In that period the patient and her husband discussed euthanasia with the general practitioner. During that conversation she indicated that she thought euthanasia was going too far. After the general practitioner explained about possible admission to a nursing home if her condition deteriorated she replied, ‘All right, maybe then.’*

*During the intake interview for the nursing home (seven weeks before her death) the husband asked the physician to perform euthanasia on the basis of the advance directive. The physician subsequently observed the patient frequently and for long periods, and spoke with her. According to the physician, she no longer understood the words ‘euthanasia’ and ‘dementia’. She regularly said to carers in the nursing home that she wanted to die. Reading between the lines, the physician concluded, on the basis of her observations and the conversations, that the patient was expressing a wish to die. But even in this period the patient’s response on several occasions when dying was discussed was, ‘Not now though, it’s not that bad yet.’*

*The physician who performed euthanasia (an elderly-care physician) twice consulted an independent SCEN physician. The first independent physician, a psychiatrist, established that the patient was decisionally incompetent and that she was suffering unbearably without prospect of improvement. According to the first independent physician the suffering consisted of having completely lost control of her life and being in a situation that she did not understand and did not want. Her life appeared to be a succession of incidents involving aggression, despair, restlessness and exhaustion. As far as this independent physician was concerned, the advance directive took the place of an oral request for euthanasia. The second independent physician also concluded that the due care criteria had been complied with.*

*The physician performed euthanasia by first administering 15mg of Dormicum dissolved in coffee (as premedication) and then after 45 minutes another 10mg of Dormicum by subcutaneous injection. Around 40 minutes later the physician administered 2000mg of thiopental intravenously, followed several minutes later by 150mg of rocuronium. In her report the physician noted that the patient awoke when the thiopental was being injected and put up physical resistance.*

*The committee asked the physician for an oral explanation. The committee noted that the patient had been admitted to a nursing home even though she had always rejected that notion. It also noted that she was no longer able to request euthanasia herself, whereas she had always assumed – according to the texts of the various advance directives – that she would be able to ask for it herself. The committee also had questions about the actual euthanasia procedure. (Initially there were also questions about her suffering, but the physician plausibly argued before the committee that she was reasonably able to conclude that the patient was suffering unbearably without prospect of improvement.)*

*As regards the request, the physician stated that she first met the patient when she was admitted to the nursing home. The patient was decisionally incompetent at the time. The physician thought that she was entitled to euthanasia due to her suffering and the fact that it was clear from her advance directive that she had never wanted to end up in a nursing home. The physician checked with the attending geriatrician and the general practitioner as to whether the patient was decisionally competent when she drew up the advance directives. Both said this was the case.*

*The physician did not take the patient’s response when the thiopental was administered as a sign that she might no longer want euthanasia. As the patient was decisionally incompetent, what she was expressing at that moment was not relevant to the physician. Nor did the physician think it would be appropriate to halt the euthanasia process at that moment.*

*The committee also put questions to the patient’s former general practitioner. These questions concerned the conversations held about euthanasia and the point at which the patient became decisionally incompetent. The general practitioner had several conversations withthe patient when she was still decisionally competent. It was clear that she did not want to go into a nursing home, but also that she felt euthanasia was not yet necessary. Later she became less clear about her wishes concerning euthanasia. When it became clear, several months before her death, that admission into a nursing home would become necessary, the general practitioner invited her and her husband to the surgery. At that time, euthanasia was not on her mind, nor did she understand what it meant any more. After the general practitioner explained the meaning of euthanasia, she said she did not want that. When reminded of her wish not to go into a nursing home, she said that then she might want euthanasia after all. When the general practitioner explained to her how it worked, she thought that was ‘going too far’. In other words, she was no longer able to indicate what her wishes were concerning euthanasia. The general practitioner was unable say exactly when the patient had become decisionally incompetent in relation to her request for euthanasia. It had happened some time in the year before her death.*

*In the interview with the committee the elderly-care specialist explained that she had administered the Dormicum dissolved in coffee because the patient was not taking any medication and she would probably have refused had she been asked to take the Dormicum herself. When it became clear that the Dormicum was having insufficient effect, the extra dose was administered. The patient did not like the needle prick. After some time had passed and it was clear the patient was unaware of what was going on around her (moving of furniture etc.) a cannula was inserted. This was difficult and took a long time, but she seemed to be unaware of it. However, when the physician tried to administer the thiopental, the patient sat up. This is what the physician had previously referred to as physical resistance. The family then held her and the physician quickly administered the rest of the euthanatic.*

*The committee found that the dementia clause written in the year prior to the patient’s death, which accompanied the advance directive, could be read in more than one way. It can be inferred from the wording (‘when I myself think the time is ripe’ and ‘at my request’), viewed in the context of the wording of the first dementia clause (‘when I am still to some degree decisionally competent’), that when the patient drew up these provisions she assumed that she would be able to request euthanasia herself when the time came and that she would indeed do so. It therefore does not follow necessarily from the text of the advance directive in conjunction with the dementia clause, as revised in the year prior to her death, that it was drawn up to take the place of an oral request in the event that she would be unable to determine or express her wishes as a result of dementia. The committee did realise that a different, wider interpretation was possible which assumed that the directive was indeed drafted to take the place of an oral request. It found, however, that the last dementia clause offered an insufficiently clear basis for such a wider interpretation. Assuming that it did leads to two mutually exclusive interpretations of the clause. In that case, doubt persists as to whether the patient wanted the advance directive to take the place of an oral request. Given this doubt, and taking into account the fact that this was literally a matter of life and death since termination of life is irreversible, in the committee’s opinion those involved should have erred on the side of caution and applied the more restrictive interpretation of the dementia clause. It follows that section 2 (2) did not apply.*

*In the absence of an oral request from the patient asking the physician to actually perform euthanasia and the absence of a clear advance directive to replace such a request, the committee found that the physician could not have concluded unequivocally that she had made a voluntary and well-considered request for euthanasia.*

*As regards the actual euthanasia procedure, the committee found that the physician’s actions overstepped a boundary. By – covertly – administering Dormicum, she wanted to deprive the patient of the possibility to resist the insertion of the cannula or the administering of the euthanatics. The committee found that, when the patient did respond negatively, the physician wrongly failed to consider whether this could be interpreted as an important sign that she did not want a cannula and a needle to be inserted. Although the committee acknowledged that it was extremely difficult for the physician to correctly interpret what the patient was expressing at that time, it found that the physician should at least have taken the time to do so. The committee considered that, although the patient was decisionally incompetent in relation to euthanasia, this did not necessarily rule out that she was able to determine her wishes with regard to actions such as inserting a cannula or a needle, even if she were no longer able to understand the purpose of those actions.*

*In the committee’s opinion the physician should have halted the euthanasia procedure in order to reconsider the current situation instead of proceeding. The committee also considered that, when performing euthanasia, coercion – and anything that might suggest coercion – must be avoided. It therefore concluded that any claim that euthanasia was performed with due medical care is untenable.*

**CASE 2017-14**

**ADVANCED-STAGE DEMENTIA**

**FINDING: due care criteria complied with**

**KEY POINTS: non-straightforward notification; disagreement among**

**specialists consulted**

*The patient, a woman in her eighties, was diagnosed three years before her death with a dementia syndrome that most closely resembled Alzheimer’s disease. Her condition was incurable. The patient had a dedicated care worker who provided advice and support, and she had moved into a care home. In the final period before her death, her dementia had reached such an advanced stage that admission to a secure, psychogeriatric ward was deemed necessary. The patient was utterly opposed to this idea, and she repeatedly threatened to jump out of the window if she was moved.*

*Her suffering consisted of increasing loss of memory and grasp of the world around her. She suffered severely from the prospect of being admitted to a secure ward and thus losing her independence. This prospect led to increased anxiety and irritability. She associated being placed in a secure ward with traumatic experiences she had gone through in the war and she did not want to lose her freedom again. Having to go into such a ward was the absolute limit for her. If that were to happen, also given her experience of close family members with dementia, she would not want to go on living.*

*Around 20 years before her death, the patient had drawn up an advance directive for the first time. Two years before her death, she drew up a new advance directive concerning her mental condition as well as her physical deterioration.*

*On the basis of his conversations with the patient, the physician established that she was very resolute in her refusal to go into a secure ward, and that she was also very resolute in her wish for euthanasia. He had been told by the head of care of the ward where she was staying that*

*the patient had already said two years previously, during her intake interview, that she would never want to go into a secure ward. The patient had discussed this regularly with her since then.*

*About a month before the patient’s death, at the physician’s request, an independent elderly-care specialist examined the patient to assess her decisional competence. According to the elderly-care specialist, the patient appeared to have no insight into her disease, prognosis and*

*disabilities. She seemed to have no oversight of the situation or insight into the relevant issues. He considered her to be decisionally incompetent in terms of overseeing complex issues and taking decisions on such issues.*

*The physician consulted an independent physician who was also a SCEN physician and a geriatric psychiatrist. According to the independent physician, the patient did not have a psychotic disorder or a mood disorder, and had a powerful need for control and independence, partly due to her traumatic war experiences. She was no longer able to understand the complexity of her situation. However, if the subject was put to her in a calm manner, she was able to indicate clearly that she wanted to retain her freedom, that she did not want to be placed in a secure ward, and that she did not want to suffer any further debilitation. At this point, she understood the situation sufficiently and was consistent in her wishes, according to the independent physician. The latter concluded that the patient was decisionally competent regarding her request for euthanasia, and her request was voluntary and well considered.*

*The committee considers that a request for termination of life from a patient suffering from progressive dementia must be responded to with even greater caution than usual. There may be doubts about whether the patient is decisionally competent, and in view of the nature of the condition, whether the request is voluntary and well considered. It may also be unclear whether the patient’s suffering is in fact unbearable.*

*In the committee’s opinion, the physician exercised sufficient caution in this case. The physician consulted an independent elderly-care specialist,* *as well as an independent physician who was also a geriatric psychiatrist. Both gave their opinion on the patient’s decisional competence. The elderly-care specialist considered her incompetent with regard to making decisions on complex issues. The independent physician, on the other hand, was of the opinion that she was decisionally competent regarding her request for euthanasia. In view of the independent*

*physician’s extensive substantiation of his opinion, compared to the more cursory substantiation given by the elderly-care specialist, and in view of the conversations the physician had with the patient, the committee found that the physician could consider the opinion of the independent physician/geriatric psychiatrist to be more convincing and that he could reasonably conclude that the patient was decisionally competent regarding her request.*

*The committee found that the physician could be satisfied that the patient’s request was voluntary and well considered and that she was suffering unbearably with no prospect of improvement. The other due care criteria were also fulfilled.*
